# Uremic Serum Alters Gene Expression Profiles and Signaling Pathway Activity in Porcine Arterial Smooth Muscle Cells

**DOI:** 10.3390/biom16091252

**Published:** 2026-08-28

**Authors:** Youyou Zheng, Kent A. Lee, Unimunkh Uriyanghai, Christine Wai, Mihaela Mocanu, Anthony Z. Yang, Huanjuan Su, Lianxia Li, Vinay A. Sudarsanam, John S. Poulton, Prabir Roy-Chaudhury, Gang Xi

**Affiliations:** 1 UNC Kidney Center, Division of Nephrology and Hypertension, Department of Medicine, The University of North Carolina at Chapel Hill, Chapel Hill, NC 27599, USA; yoyoz@unc.edu (Y.Z.); kentlee@unc.edu (K.A.L.);; 2 W.G. (Bill) Hefner Salisbury Department of Veterans Affairs Medical Center, Salisbury, NC 28144, USA

**Keywords:** arterial smooth muscle cells, uremia, bulk RNA sequencing, CKD, ESKD

## Abstract

Uremic conditions are common in end-stage kidney disease (ESKD) patients. Accelerated vascular diseases in uremic patients lead to heart failure, stroke, and hypertension. To investigate the effects of uremia on porcine arterial smooth muscle cells (aSMCs), bulk RNA sequencing was used to identify uremia-induced alterations in signaling pathways of aSMCs that might explain the aggressive cardiovascular diseases seen in patients with chronic kidney disease (CKD) and ESKD. Bulk RNA sequencing was performed on porcine aSMCs cultured with serum from normal or uremic pigs. Differentially expressed gene (DEG) analysis revealed that 295 genes were upregulated and 138 genes were downregulated after uremic serum exposure. Gene Ontology molecular function analysis demonstrated that ATP-dependent activity, translation factor activity, and ATP-dependent protein folding chaperones were predicted to be negatively enriched after uremic serum exposure, while proton transmembrane transporter activity, antioxidant activity, and glutathione peroxidase activity were predicted to be positively enriched. Gene set enrichment analysis indicated that the cell cycle was predicted to be negatively enriched after uremic serum exposure in aSMCs. Overrepresentation analysis found that focal adhesion, protein processing in the endoplasmic reticulum (ER) and cell senescence were predicted to be negatively enriched, while lysosome, phagosome, apoptosis, and autophagy were predicted to be positively enriched after uremic serum exposure. This study suggests that the signaling pathways that regulate cellular redox homeostasis, the cellular waste disposal system, ER stress and autophagy are major signaling pathways involved in aSMCs’ responses to uremic serum exposure. These pathways may contribute to the severe arterial-specific clinical symptoms observed in CKD/ESKD patients, such as arterial stiffness, vascular calcification and cardiovascular disease.

## 1. Introduction

Chronic kidney disease (CKD) is an increasingly common disease. In the United States alone, more than 37 million people (1 in 7 US adults) have CKD, based on the latest data from the National Kidney Foundation. Early-stage CKD has few symptoms and is hard to detect, while end-stage kidney disease (ESKD) patients have increasing morbidity and mortality. Symptoms of ESKD include nausea, loss of appetite, pain, fatigue, and weakness. Complications of CKD include high blood pressure, cardiovascular diseases (CVDs), anemia, an increased infection risk, and fluid mismanagement/imbalance [1]. CVD is the single most important cause of death in ESKD patients, accounting for more than 50% of deaths, with a mortality rate up to 20 times higher than in the general population [2]. Patients with ESKD also have high rates of coronary artery disease and heart failure [3]. In addition, CVDs in ESKD patients normally respond poorly to conventional therapies, such as statins [4]. Furthermore, a previous study demonstrated that young adult (18–40 years old) CKD patients on dialysis face increased risk of CVD death, which may be comparable to much older adults in the general population [5].

One of the primary metabolic changes caused by CKD is uremia, the elevation of urea and other nitrogenous wastes due to dysfunction of the kidneys [6]. Within the vascular system, uremia drives significant pathological changes, including vascular calcification, endothelial dysfunction, and the remodeling of vascular smooth muscle cells (VSMCs). VSMCs play a critical role in maintaining the structural and functional integrity of arteries. Under pathological conditions, such as CKD, the cells may undergo changes to synthetic phenotypes, leading to arterial stiffness, calcification, and increased cardiovascular risk [7]. Most previous investigations of uremic conditions utilized candidate gene approaches based on known protein functions to examine potential differences between normal and uremic conditions. Therefore, a more comprehensive approach is necessary to identify the differences in novel proteins or signaling pathways associated with uremia.

Alterations in gene expression under uremic conditions can help reveal the complex molecular mechanisms driving disease progression and identify potential therapeutic targets. However, a comprehensive understanding of differentially expressed genes (DEGs) in uremic arteries remains limited. A previous study in human blood cells revealed that mRNA processing and transport, protein transport, chaperone functions, and the unfolded protein response were prominently lower in uremia, while insulin-like growth factor activity, the complement system, lipoprotein metabolism and lipid transport were higher in uremia [8]. In another study, RNA was isolated from severe calcified rat aorta in chronic uremia, which showed significant enrichment of dysregulated genes for ontologies related to extracellular matrix, response to wounding, and ossification. The individually affected genes were relevant to osteogenic transformation, tissue calcification, and Wnt modulation [9]. Unfortunately, no RNA sequencing study has focused on the effect of uremic toxins on VSMCs. Understanding the pathways affected in uremic conditions contributes to broader strategies aimed at mitigating vascular complications in CKD patients, thereby reducing the morbidity, mortality, and costs of the disease. Because the major vascular diseases associated with uremic conditions, such as atherosclerosis and calcification, mainly occur in arteries, we investigated the effects of uremia on gene expression in arterial smooth muscle cells (aSMCs). We chose a porcine model because they are anatomically and physiologically more similar to humans than small rodents.

## 2. Material and Methods

### 2.1. Cell Culture and RNA Isolation

The animal study protocol was approved by the University of North Carolina at Chapel Hill IACUC (Chapel Hill, NC, USA) (IACUC ID: 22069 on 28 April 2022). The pigs were housed at the UNC–Chapel Hill animal facility, which aligns with IACUC and USDA regulations that prioritize social interaction and animal welfare. Porcine aSMCs were isolated from the carotid artery of a 4-month-old female Yorkshire pig (62 kg). The cells were cultured with growth medium (DMEM containing 5 mM glucose and 30% normal pig serum or 30% uremic pig serum plus 1% penicillin/streptomycin). Uremic pig serum was obtained via a procedure described previously, and uremic toxin values were determined [10]. Briefly, two 4-month-old male Yorkshire Cross pigs (50–60 pounds) underwent unilateral nephrectomy followed by selective ligation of the arterial tree of the contralateral kidney in order to obtain 7.5% of the contralateral kidney with viable perfusion. Uremic serum was obtained from these two uremic pigs after creatinine levels reached >8 mg/dL for over 3 weeks. The biochemical parameters and uremic toxin levels were measured (Supplemental Tables S1 and S2). Because the values were similar, one uremic serum sample (blood urea nitrogen 43 mg/dL and creatinine 9.5 mg/dL) was used in this study. Control serum was obtained from an age-matched male non-uremic pig. All experiments were performed on cells at passage 6. When cells reached 100% confluency, total RNA from 3 individual plates of each treatment (after 120 h uremic pig serum or normal pig serum exposure) was isolated using a RNeasy plus mini kit (Qiagen, Germantown, MD, USA) for bulk RNA sequencing (Novogene, Sacramento, CA, USA).

### 2.2. Library Construction, Quality Control and Sequencing

The workflow of the project is presented in Supplemental Figure S1. Briefly, messenger RNA was purified from total RNA using poly-T oligo-attached magnetic beads. After fragmentation, the first-strand cDNA was synthesized using random hexamer primers, followed by second-strand cDNA synthesis using either dUTP for directional library or dTTP for non-directional library. The library was checked with Qubit and real-time PCR for quantification and bioanalyzer for size distribution detection. Quantified libraries were pooled and sequenced on the Illumina platform according to effective library concentration and data amount.

### 2.3. Data Analysis and Statistical Analysis

**Figure 1:**
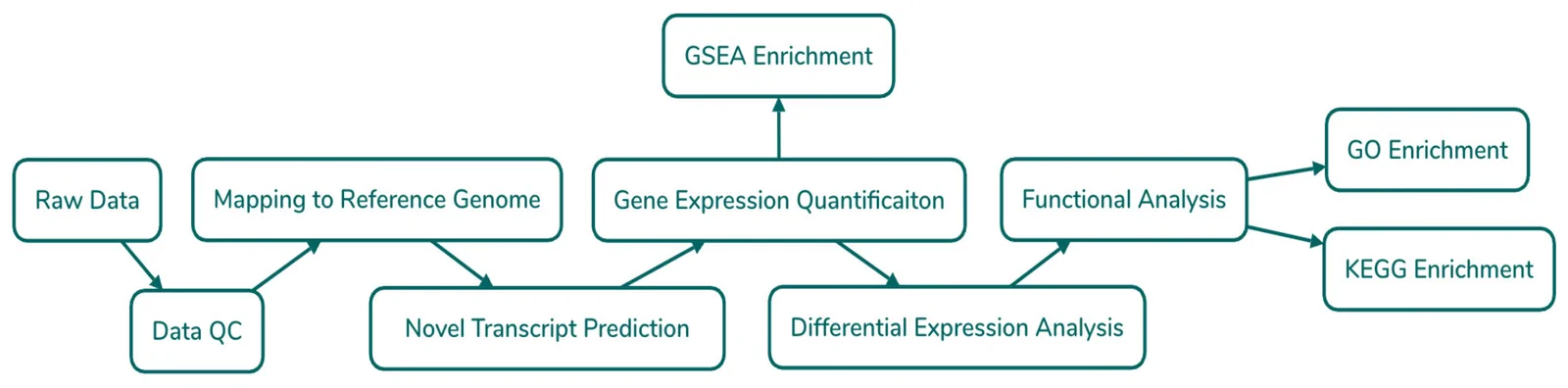
A scheme of bulk RNA sequencing data analysis.

Bulk RNA sequencing data were analyzed following the flowchart in Figure 1. Briefly, sequenced reads/raw reads often contain low-quality reads with adapters, which affect analysis quality and reliability. To avoid this, raw reads filtering is performed to remove reads containing adapters, to remove reads containing *N* > 10% and to remove the reads that have a quality value where over 50% of the bases of the read are <=5. Reference genome and gene model annotation files were downloaded from the genome website directly. Index of the reference genome was built using Hisat2 (v2.0.5), and paired-end clean reads were aligned to the reference genome using Hisat2 (v2.0.5). The mapped reads of each sample were assembled by StringTie (v1.3.3b) in a reference-based approach. FeatureCounts (v1.5.0-p3) was used to count the read numbers mapped to each gene. The expected number of Fragments Per Kilobase of transcript sequences per Millions base pairs sequenced (FPKM) of each gene was calculated based on the length of the gene and read count mapped to this gene.

#### 2.3.1. Differential Expression Analysis and DEG Definition

Differential expression results for uremic pig serum-treated aSMCs (UPA) vs. normal pig serum-treated aSMCs (NPA) were summarized as log2 fold change (log_2_FC; computed as UPA/NPA) with corresponding nominal *p*-values and Benjamini–Hochberg adjusted *p*-values (FDR). Genes were considered differentially expressed for visualization using the thresholds |log_2_FC| ≥ 1 and FDR (padj) < 0.05.

#### 2.3.2. Heatmap Visualization of DEGs

For heatmap visualization, genes meeting the DEG cutoff were plotted across the six samples (NPA1–3, UPA1–3). Expression values were standardized per gene (row-wise scaling; z-score) to emphasize relative expression patterns across samples, and hierarchical clustering was performed for both genes and samples using expression similarity.

#### 2.3.3. Gene Ontology (GO) Molecular Function Enrichment (Up vs. Down)

GO enrichment was performed separately for UPA-upregulated and UPA-downregulated gene sets using a hypergeometric framework with multiple-testing correction by the Benjamini–Hochberg method (FDR control). Raw enrichment *p* < 0.05 and FDR/q < 0.10 (as labeled on the figure) were used for significance filtering. Dot size represents the number of genes annotated to each term (“Count”), and color encodes adjusted *p*-value.

#### 2.3.4. KEGG Pathway Enrichment Overrepresentation Analysis (ORA) and Gene Set Enrichment Analysis (GSEA)

KEGG enrichment was evaluated using two complementary strategies:

- ORA—Enriched pathways were identified from UPA-up and UPA-down DEGs lists using a hypergeometric test with Benjamini–Hochberg correction.
- GSEA—Genes were pre-ranked by log_2_FC (UPA/NPA) and tested for coordinated pathway shifts across the full ranked list.

In the plots, the x-axis shows GeneRatio (fraction of genes contributing to the signaling pathway under the plotted definition), dot size indicates the number of contributing genes (“Count”), and color indicates multiple-testing-adjusted significance (padj). For GSEA, pathways are separated into activated vs. suppressed based on the sign of the enrichment direction (positive vs. negative enrichment in UPA relative to NPA).

#### 2.3.5. KEGG Pathway Map Visualization

Selected pathways were visualized by projecting gene-level log_2_FC values onto KEGG pathway schematics using Pathview. In pathway maps, node fill color represents log_2_FC (UPA/NPA): red indicates higher expression in UPA, and green indicates lower expression in UPA (relative to NPA).

## 3. Results

### 3.1. General Profiles of DEGs in NPA and UPA

**Figure 2:**
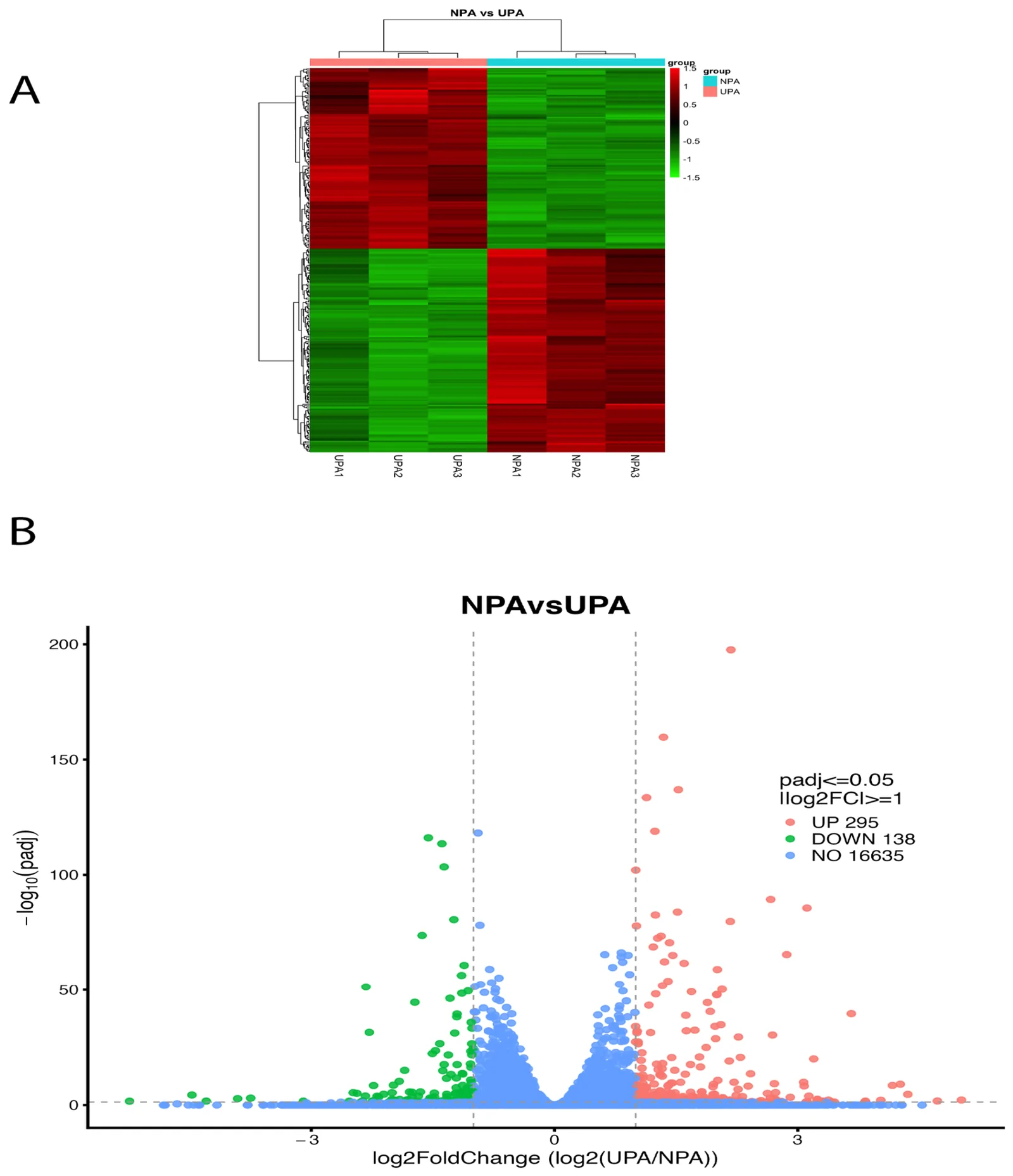
Global transcriptomic differences between NPA and UPA. (**A**) Heatmap of differentially expressed genes (DEGs) between NPA and UPA across six samples (NPA1–3, UPA1–3). Genes shown meet the cutoff |log_2_FC (UPA/NPA)| ≥ 1 and padj < 0.05. Values are scaled per gene (row-wise z-score) to highlight relative expression patterns. Both genes and samples are hierarchically clustered. (**B**) Volcano plot of all tested genes showing log_2_FC (UPA/NPA) versus −log10(padj). Dashed lines indicate the DEG cutoffs (|log_2_FC| ≥ 1, padj < 0.05). Pink points indicate significantly upregulated genes in UPA; green points indicate significantly downregulated genes in UPA; blue points indicate non-significant changed genes.

aSMCs were isolated from the pig carotid artery and cultured in normal pig serum-or uremic pig serum-containing growth media before RNA isolation. To explore the general profiles of DEGs in NPA or UPA, we first created a heatmap of the top 300 DEGs across these two groups with the cutoff of padj < 0.05 (Figure 2A). Columns represent different samples within each group, while rows correspond to individual genes. Gene expression levels are color-coded: red indicates upregulation and green indicates downregulation after UPS exposure. The two treatment groups showed clearly different patterns of DEGs.

To examine the variability in gene expression of aSMCs after uremic serum exposure, we also generated a volcano plot of DEGs displaying log_2_ fold change (x-axis) versus –log_10_ (adjusted *p*-value) (y-axis) for all genes assessed in the DEG analysis (Figure 2B). Vertical and horizontal dotted lines represent the significance thresholds: |log_2_(fold change)| ≥ 1 and adjusted *p*-value ≤ 0.05. Red or green points denote DEGs that were significantly upregulated or downregulated in aSMCs after pig uremic serum exposure, respectively. Blue points denote no significant changes in gene expression. Under the current cutoff, 295 genes were upregulated and 138 genes were downregulated after uremic serum exposure compared to normal pig serum exposure. A total of 16,635 gene expressions were not significantly changed after uremic serum exposure.

### 3.2. Functional Enrichment Analysis

**Figure 3:**
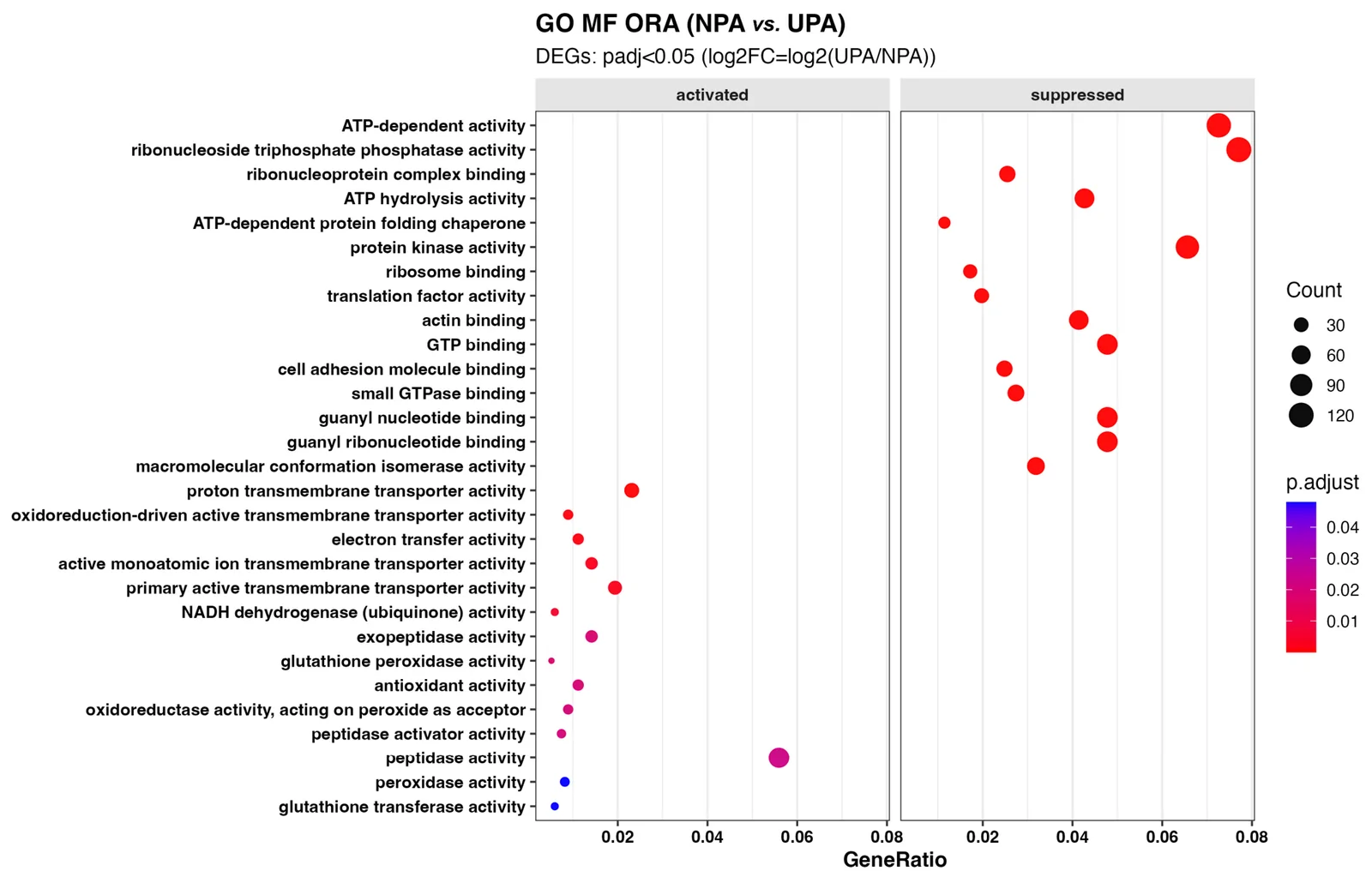
GO MF enrichment analysis in UPA-up vs. UPA-down DEG. Dot plots of enriched GO MF terms for DEGs separated by direction: UPA-upregulated (**left**) and UPA-downregulated (**right**). Enrichment significance thresholds are raw *p* < 0.05 and FDR/q < 0.10 (as labeled). The x-axis shows GeneRatio (fraction of the input gene list annotated to the term). Dot size indicates the number of genes mapping to each term (Count), and dot color indicates adjusted significance (padj).

GO molecular function (MF) enrichment analysis was performed using clusterProfiler (version 4.18.4). A dot plot of GO MF terms enriched among DEGs (DEGs padj < 0.05; GO *p* < 0.05, q < 0.1) was generated using ggplot2 (version 4.0.1) (Figure 3). The y-axis lists the enriched GO MF terms, and the x-axis (GeneRatio) is the fraction of input genes annotated to each term (a larger GeneRatio means that a larger share of the DEG list maps to that GO term). Color gradient represents adjusted *p*-values (red: lower, blue: higher), and dot size reflects the number of annotated DEGs in each category. The results revealed that genes in some categories were downregulated after uremic serum exposure, such as ATP-dependent activity (e.g., *MYO1D*, *HSPA2* and *DDX21*), ATP hydrolysis activity (e.g., *HSP90AA1*, *ATP2B4* and *KIF5B*), ATP-dependent protein folding chaperone (e.g., *HSPA2*, *HSPA9* and *HSPA5*), protein kinase activity (e.g., *PDK1*, *PGK1* and *BMPR2*), translation factor activity (e.g., *EEF2*, *EIF3A* and *EIF4G1*) and GTP binding (e.g., *RASL12*, *EHP2* and *SEPTIN7*) (Figure 3 left). The inhibition of ATP-dependent activity is a cellular response to stress conditions, which is vital for cell survival [11]. However, the suppression of functional ATP-dependent chaperones leads to the accumulation of misfolded proteins, irreversible protein aggregation and increased susceptibility to diseases. Meanwhile, several categories were transcriptionally upregulated after uremic serum exposure in aSMCs, such as proton transmembrane transporter activity (e.g., *ATP8*, *ATP6V0C* and *COX1*), electron transfer activity (e.g., *NDOR1*, *COX3* and *CYTB*), glutathione peroxidase activity (e.g., *GPX1*, *PTGES* and *GPX4*), antioxidant activity (e.g., *SOD3*, *PTGE* and *GPX4*) and glutathione transferase activity (e.g., *PTGES*, *MGST1* and *GSTK1*) (Figure 3 right). The activation of proton and electron transfer activities indicates the initiation or acceleration of redox processes, which lead to reactive oxygen species (ROS) generation. Indeed, genes associated with cellular antioxidant activities were also upregulated, presumably to counter oxidative stress.

**Figure 4:**
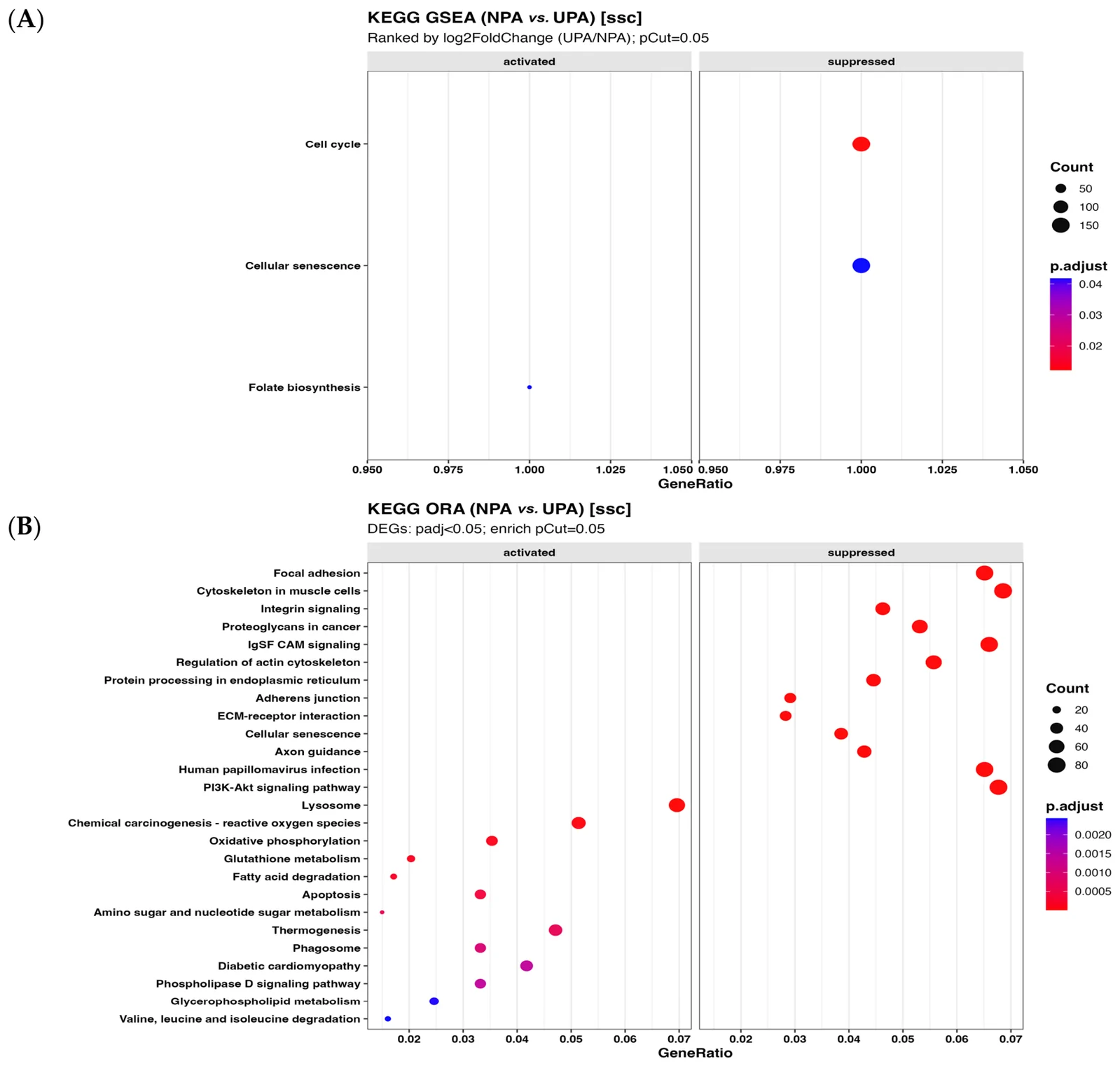
KEGG pathway enrichment by GSEA and ORA in NPA vs. UPA. (**A**) KEGG GSEA dot plot using genes ranked by log_2_FC (UPA/NPA). Pathways are separated into activated vs. suppressed based on enrichment direction in UPA relative to NPA. Dot size indicates the number of genes contributing to the enrichment signal, and color indicates padj. (**B**) KEGG ORA dot plot showing pathways enriched among UPA-up and UPA-down DEG sets (DEGs defined at padj < 0.05; enrichment pCut as labeled). The plot is formatted to match the GSEA style: pathways are separated by direction, dot size reflects count, and dot color encodes padj.

Functional pathway enrichment was also performed by GSEA (ranked by log_2_Foldchange; pCut = 0.05) and ORA (padj < 0.05; enrich pCut = 0.05) using DEGs from the NPA vs. UPA comparisons (Figure 4). The GSEA results indicated that cell cycle-associated genes were downregulated and folate biosynthesis was transcriptionally enriched after uremic serum exposure in aSMCs (Figure 4A). Meanwhile, the ORA results predicted that several signaling pathways were negatively enriched, such as focal adhesion, cytoskeleton in muscle cells, integrin signaling, the regulation of actin cytoskeleton, protein processing in endoplasmic reticulum and cell senescence. By contrast, lysosome, phagosome, apoptosis and oxidative phosphorylation were predicted to be positively enriched after uremic serum exposure (Figure 4B).

### 3.3. KEGG Pathways Mapping of DEGs

To put the specific changes in DEGs in the context of a specific pathway, DEGs obtained in this study were projected onto the relevant KEGG pathways known to play crucial roles in cell behavior and biological function. Gene-level log_2_FC values from DEGs were projected onto pathway diagrams using the Pathview KEGG database [12,13]. In pathway maps, node fill color represents log_2_FC (UPA/NPA): red indicates higher expression under UPA, and green indicates lower expression under UPA (relative to NPA).

#### 3.3.1. Uremic Serum Exposure Alters Gene Expression in Pathways Regulating the Cell Cycle and Cellular Senescence

**Figure 5:**
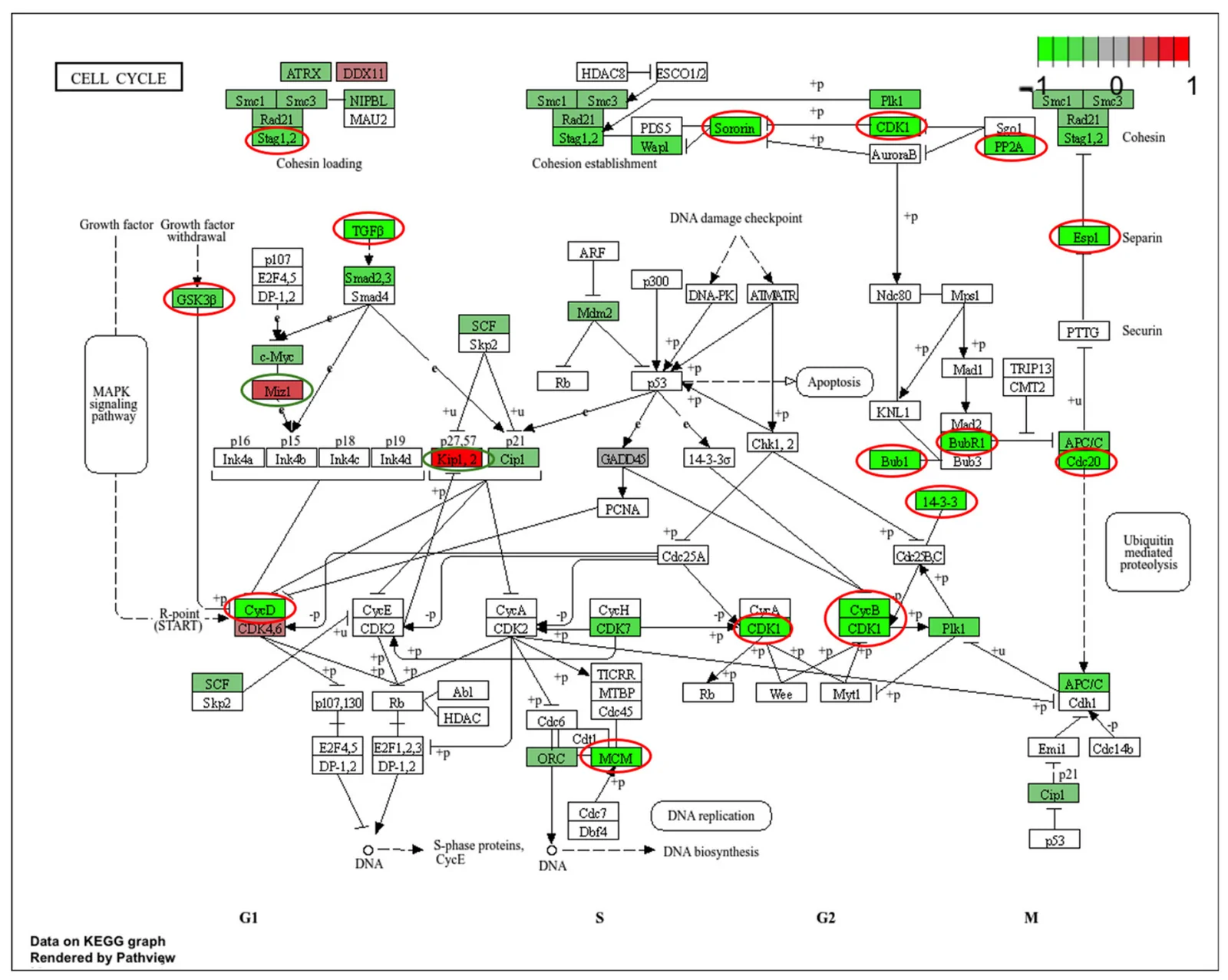
Cell cycle signaling pathway map. KEGG cell cycle signaling pathway with DEG log_2_FC values overlaid (UPA/NPA). Nodes are colored by log_2_FC (red: higher in UPA; green: lower in UPA). Grey/white nodes indicate genes not present in the DEG input or not mapped in the pathway. Solid arrows represent direct regulatory interactions. Dotted arrows indicate an indirect effect. Dotted lines represent interactions without a directional effect. Red and green circles indicate the genes that were mentioned in the text.

Our KEGG GSEA analysis identified that the cell cycle pathway was predicted to be negatively enriched after uremic serum exposure. In the cell cycle pathway (Figure 5), downregulated genes in aSMCs exposed to uremic serum included *GSK3B*, *STAG1/2*, *TGFB*, *CCND* (*CycD*), *CCNB* (*CycB*), *CDK1*, *CDCA5* (*Sororin*), *PP2A*, *BUB1* (*Bub1*), *BUB1B* (*BubR1*), *CDC20*, *YWHA* (*14-3-3*), *Esp1* and *MCM*, while cyclin-dependent kinase inhibitor *CDKN1B/1C* (*Kip1/2*) and cell cycle inhibitor *ZBTB17* (*Miz1*) were upregulated after exposure to uremic serum, which further inhibit the cell cycle.

Because our KEGG ORA analysis showed that cellular senescence was predicted to be negatively enriched after uremic serum exposure, the DEGs were projected onto this pathway, which revealed that *TGFB*, *TGFBR*, *MEK*, *IL1A*, *HIPK2*, *CCND* (*CycD*), *CDK1*, *CCNB* (*CycB*), *PP1A* and *SERPINE1* (*PAI-1*) expression were downregulated whereas *HLA-G*, *MAPK14* (*p38*), *TSC1/2*, and *IL6* expression were upregulated after uremic serum exposure (Supplemental Figure S3).

#### 3.3.2. Uremic Serum Exposure Alters Gene Expression in Pathways Regulating Focal Adhesion and Cytoskeleton Structure

**Figure 6:**
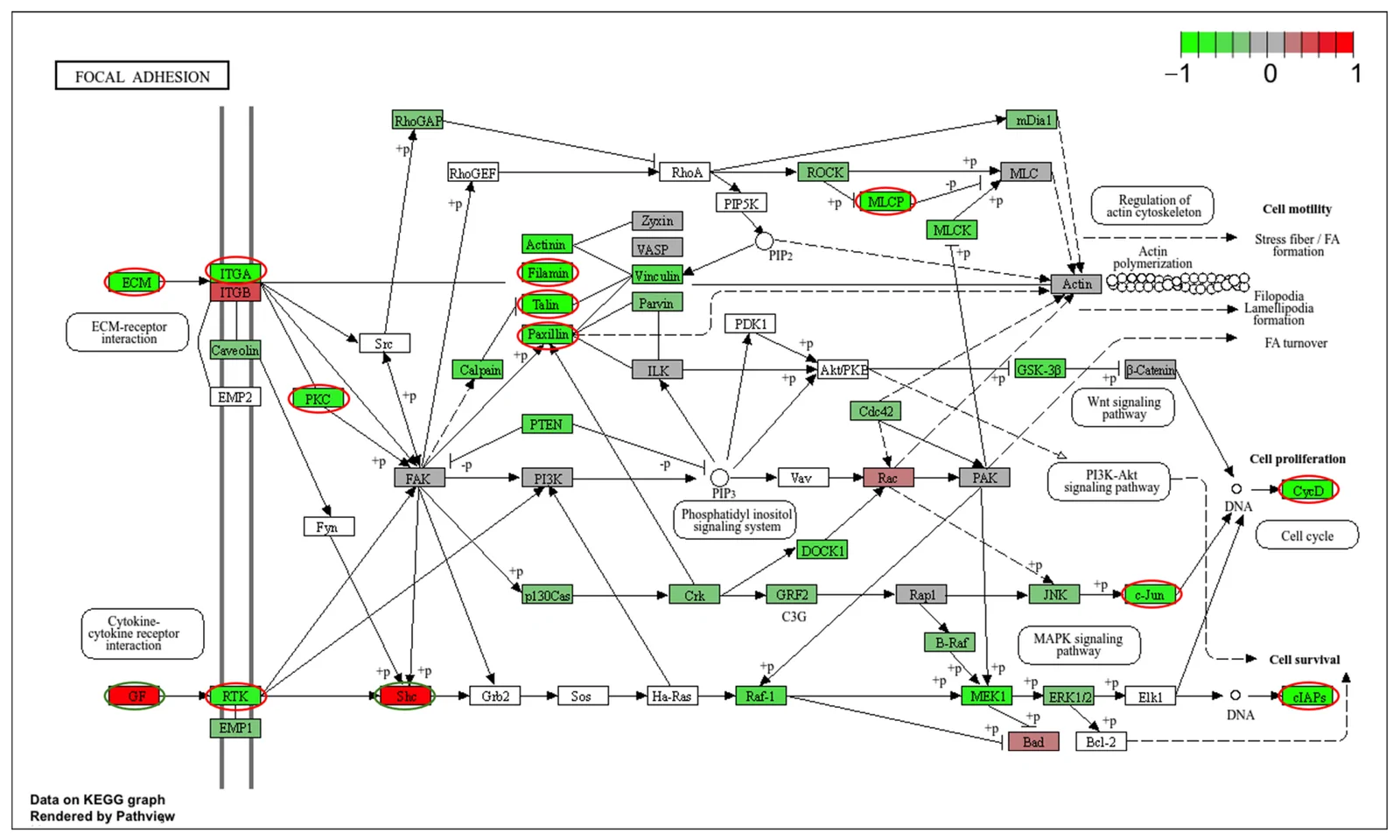
Focal adhesion pathway map. KEGG focal adhesion pathway with DEG log_2_FC values overlaid (UPA/NPA). This map highlights coordinated changes in focal adhesion-associated signaling modules. Red indicates upregulation in UPA, and green indicates downregulation in UPA. Solid arrows represent direct regulatory interactions. Dotted arrows indicate an indirect effect. Red and green circles indicate the genes that were mentioned in the text. DEG data projected on the cytoskeleton in muscle cells pathway showed the downregulation of *COL*, *ELM*, *FBN*, *ITGA*, *DES*, *TLN*, *SGCB*, *DES*, *MYH*, *ACTA/C*, *SNTA/B*, *FLNC*, *PDLIM*, *ANKRD*, *CAPZ*, *FMN* (*Formin*), *SYNPO2*, and *SYNE3*. Meanwhile, a set of genes were upregulated, including *DCN*, *SGCG/Z*, *TNNI3*, *MYOM* and *OBSCN*, after uremic serum exposure (Supplemental Figure S4). Another cytoskeletal pathway that was predicted to be negatively enriched by exposure to uremic serum was the regulation of the actin cytoskeleton pathway. The analysis results demonstrated that a large group of genes were downregulated, including *F2R*, *ITG*, *GPCR*. *RGC32*, *Raf*, *MEK*, *IQGAP*, *Drf3*, *PXN*, *ERM*, *ACTN*, *DIAPH* (*mDia*), *MLCP*, and *Myosin II*. Meanwhile, a small group of genes were upregulated, such as *CXCL12*, *GF*, *C5b-9* and *LPAR* (Supplemental Figure S5).

Cellular structural integrity and contractile function are important features of VSMCs. Differences in focal adhesion, cytoskeletal dynamics and extracellular matrix (ECM) interactions help adapt to the mechanical demands of arterial vascular environments. Our KEGG ORA analysis revealed that multiple related pathways were predicted to be negatively enriched after uremic serum exposure (Figure 4B). DEG data projected on the focal adhesion pathway (Figure 6) showed downregulation of a group of genes, including *ECM*, *ITGA*, *RTK*, *PKC*, *ACTN* (*Actinin*), *FLN* (*Filamin*), *TLN* (*Talin*), *PXN* (*Paxillin*), *MLCP*, *JUN* (*cJun*), *CCND* (*CycD*), and *BIRC* (*cIAPs*), whereas a small group of genes, such as GF and SHC, were upregulated after uremic serum exposure. In addition, a group of genes were downregulated in the integrin signaling pathway, including *COL* (*Collagen*), *LAM* (*Laminin*), *ITGA1*, *ITGA3*, *THBS*, *ITGA7*, *OPN*, *TNC* (*Tenascin*), *FBN1*, *NpnT*, *ITGA*, *FLN* (*Filamin*), *TTLN* (*Talin*), *PXN* (*Paxillin*), *ACTN* (*Actinin*), *LIM* (*Pinch*) and *CCND* (*CycD*). Only a small group of genes in this pathway were upregulated after uremic serum exposure, including *ITGA10*, *ITGB4*, *VCAM1*, *DPP*, *VTN* (*vitronectin*), *DMP1*, *DSP* and *GF* (Supplemental Figure S2).

#### 3.3.3. Uremic Serum Exposure Alters Gene Expression in Pathways Regulating Protein Post-Translational Processing and Cellular Waste Disposal

**Figure 7:**
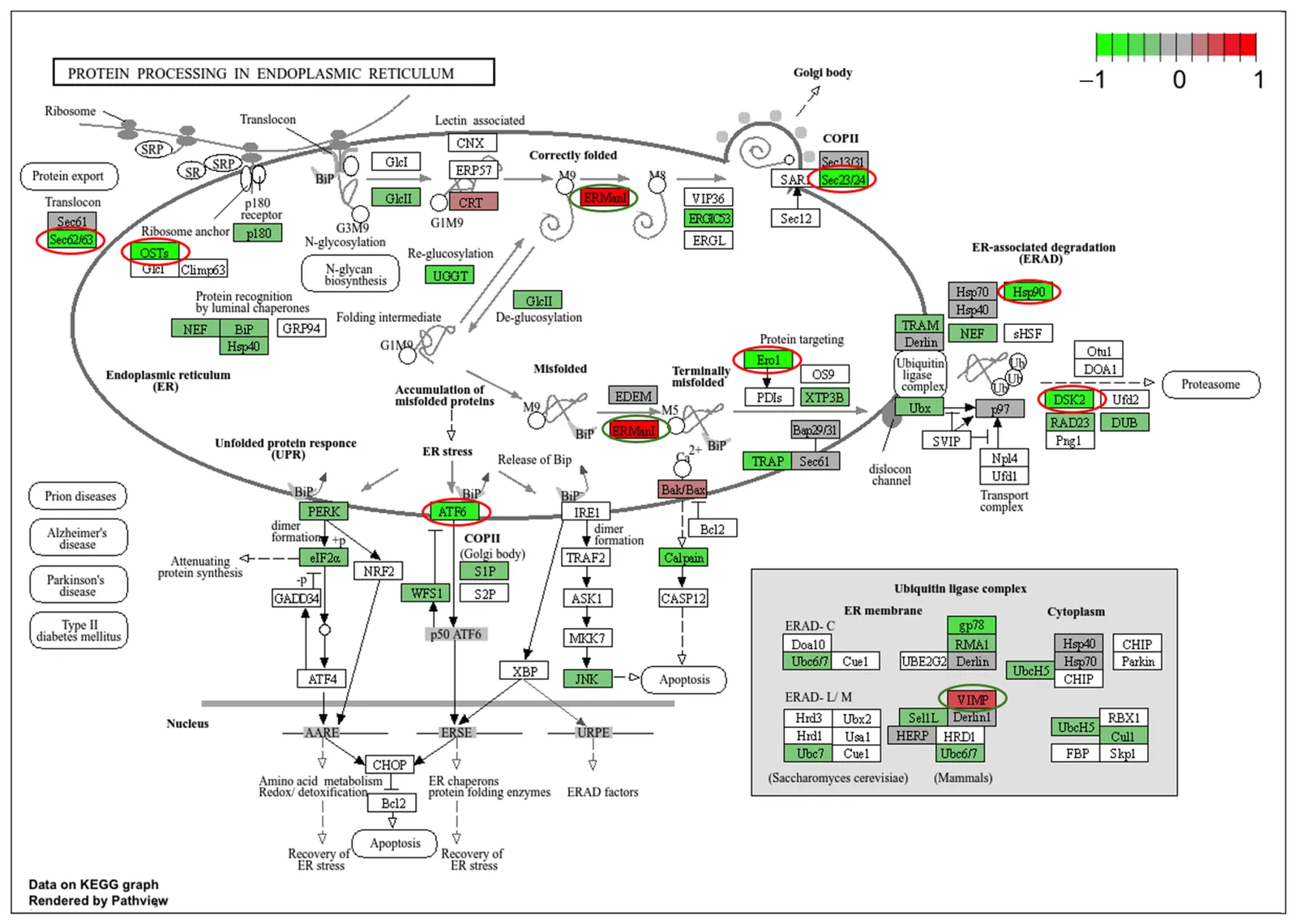
Protein processing in endoplasmic reticulum pathway map. KEGG protein processing in endoplasmic reticulum pathway with DEG log_2_FC values overlaid (UPA/NPA). Red indicates upregulation in UPA, and green indicates downregulation in UPA. Solid arrows represent direct regulatory interactions. Dotted arrows indicate an indirect effect. Red and green circles indicate the genes that were mentioned in the text.

The KEGG ORA analysis revealed that protein processing in the endoplasmic reticulum (ER) was predicted to be negatively enriched in aSMCs after uremic serum exposure (Figure 4B). DEGs projected onto this specific pathway identified a group of genes that were downregulated, including *Sec62/63*, *OSTs*, *ATF6*, *Sec23/24*, *Ero1*, *Hsp90*, and *DSK2,* whereas only a few genes, such as *MAN1B1* (*ERManI*) and *VIMP*, were upregulated (Figure 7). In general, cell stress is thought to activate the unfolded protein response (UPR) to stop further protein production [14], which may explain our observation of the suppression of protein processing in the ER in response to uremic serum exposure. Indeed, elevated ER stress has been associated with CKD patients [15].

**Figure 8:**
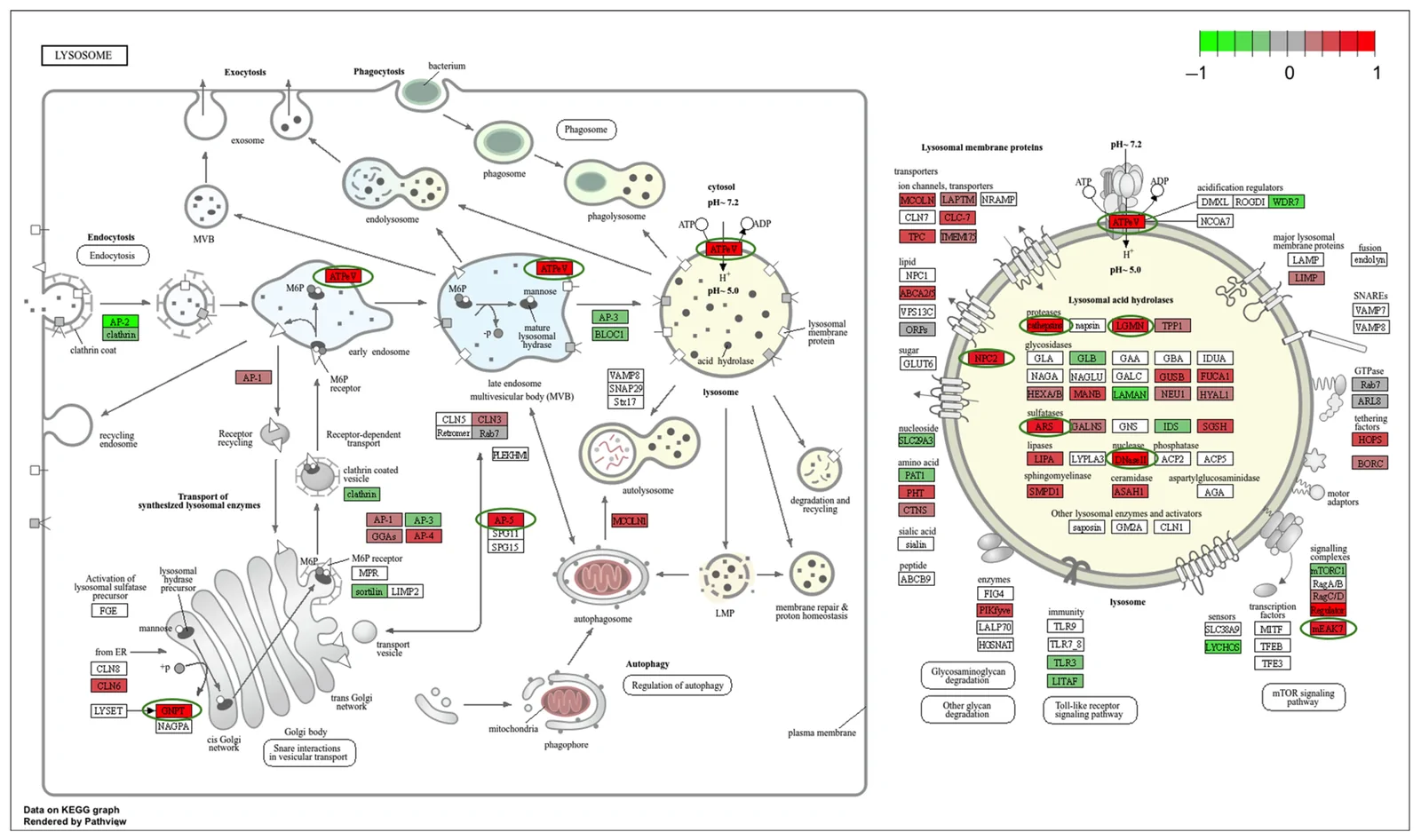
Lysosome pathway map. KEGG lysosome pathway with DEG log_2_FC values overlaid (UPA/NPA), summarizing altered lysosome signaling pathway components. Red indicates upregulation in UPA, and green indicates downregulation in UPA. Solid arrows represent direct regulatory interactions. Green circles indicate the genes that were mentioned in the text.

Meanwhile, the KEGG ORA analysis revealed that several pathways associated with the cellular waste disposal system, which plays a crucial role in maintaining normal cellular physiological functions [16], were predicted to be positively enriched. Lysosomes are a major component of cellular waste processing, and our KEGG ORA analysis indicated that several lysosome pathway-associated genes were upregulated after uremic serum exposure, which included *ATPeV*, *GNPT*, *AP5*, *CTS* (*Cathepsins*), *LGMN*, *NPC2*, *ARS*, *DNASE2* (*DNaseII*) and *MEAK7* (Figure 8). In addition, phagosome, apoptosis and autophagy pathways were also predicted to be positively enriched under uremic conditions, which indicates a multifaceted enhancement of cellular waste disposal.

#### 3.3.4. Uremic Serum Exposure Alters Gene Expression in Pathways Regulating Cellular Redox Homeostasis

**Figure 9:**
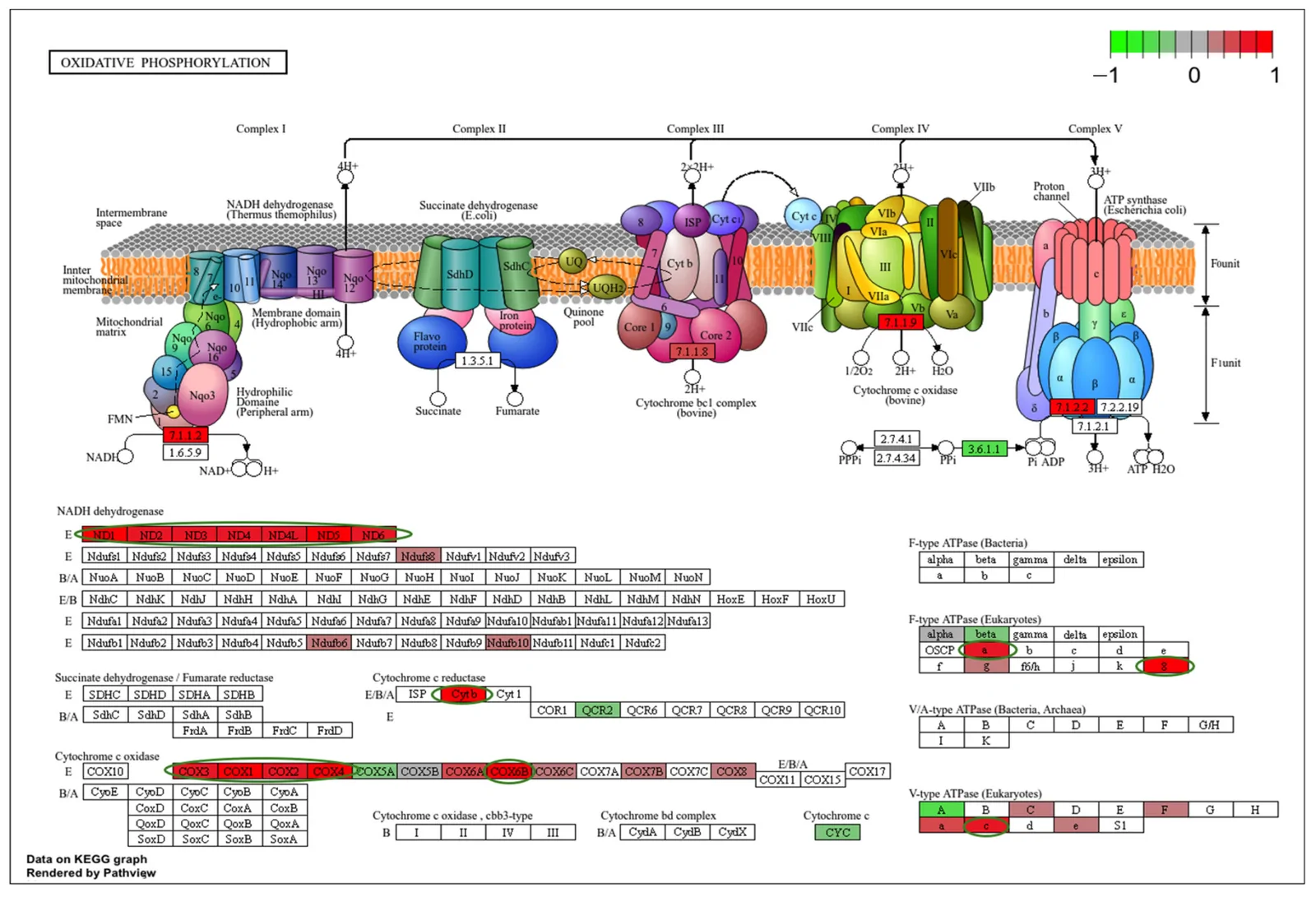
Oxidative phosphorylation pathway map. KEGG oxidative phosphorylation pathway with DEG log_2_FC values overlaid (UPA/NPA). Red indicates upregulation in UPA, and green indicates downregulation in UPA, summarizing altered oxidative phosphorylation signaling components. Solid arrows represent direct regulatory interactions. Dotted arrows indicate an indirect effect. Green circles indicate the genes that were mentioned in the text.

Consistent with the GO MF ORA analysis (Figure 2), the KEGG ORA analysis revealed that several pathways that regulate cellular redox homeostasis were predicted to be positively enriched after uremic serum exposure (Figure 3 left). These pathways included oxidative phosphorylation, glutathione metabolism, fatty acid degradation (β-oxidation), and peroxisome signaling pathways. DEGs projected onto the oxidative phosphorylation pathway identified a group of genes that were upregulated, including *ND1-6*, *Cytb*, *COX1-4*, *COX6B* and *ATP5F1Z* (*F-type ATPase ζ*) (Figure 9).

**Figure 10:**
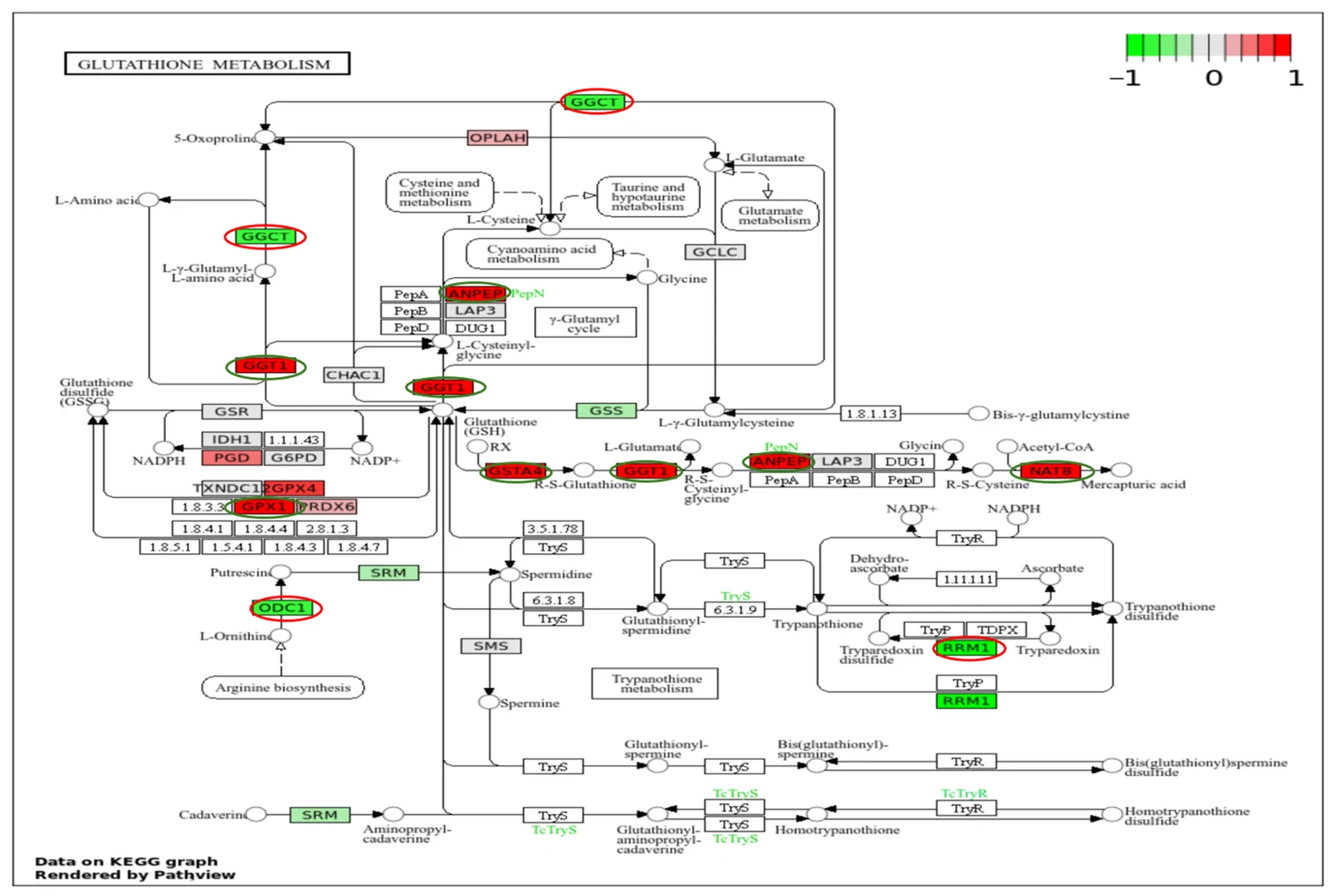
Glutathione metabolism pathway map. KEGG glutathione metabolism pathway with DEGs log_2_FC values overlaid (UPA/NPA). Red indicates upregulation in UPA and green indicates downregulation in UPA, summarizing altered glutathione metabolism signaling components. Solid arrows represent direct regulatory interactions. Dotted arrows indicate an indirect effect. Red and green circles indicate the genes that were mentioned in the text.

The activation of glutathione metabolism normally enhances cellular antioxidant defense and detoxification by increasing reduced glutathione (GSH), which prevents cellular oxidative damage by scavenging free radicals and ROS [17]. The DEG after uremic serum exposure revealed a group of upregulated genes, including *GGT1*, *ANPEP*, *GPX1*, *GSTA4* and *NAT8*, whereas the downregulated genes were *GGCT*, *ODC1* and *RRM1* (Figure 10).

**Figure 11:**
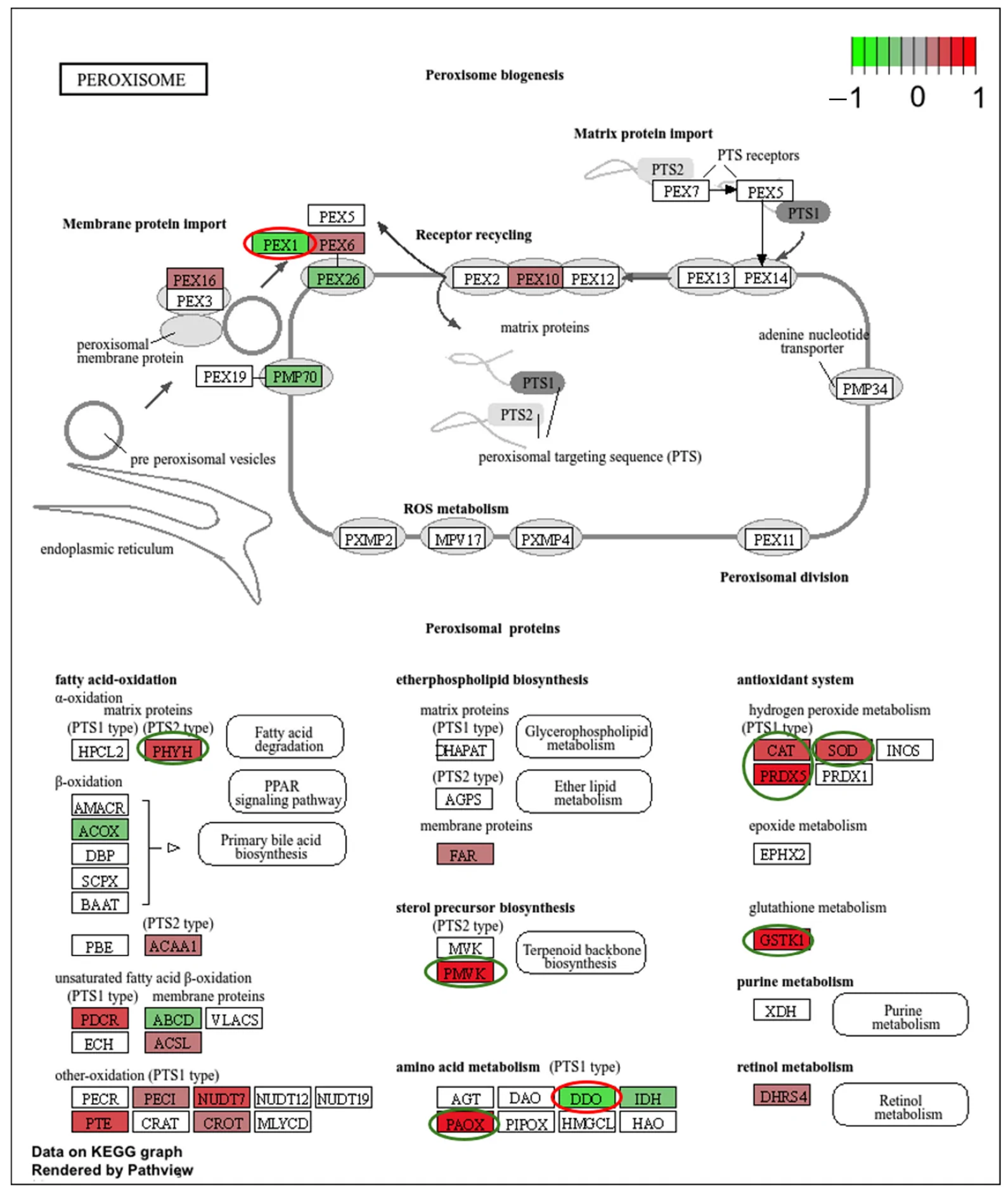
Peroxisome pathway map. KEGG peroxisome pathway with DEG log_2_FC values overlaid (UPA/NPA). Red indicates upregulation in UPA, and green indicates downregulation in UPA, summarizing altered peroxisome signaling components. Solid arrows represent direct regulatory interactions. Red and green circles indicate the genes that were mentioned in the text.

The peroxisome signaling pathway regulates cellular metabolism and redox balance. The activation of this pathway produces hydrogen peroxide during fatty acid oxidation, which is also activated during uremic conditions. Hydrogen peroxide serves as a signaling molecule to affect cellular redox, metabolic and inflammatory pathways. The upregulated genes in this pathway included *PHYH*, *PMVK*, *PAOX*, *PRDX5*, *CAT*, *SOD* and *GSTK1*, whereas the downregulated genes were *PEX1* and *DDO* (Figure 11).

#### 3.3.5. Uremic Serum Exposure Alters Gene Expression in Pathways Regulating Osteogenesis

Vascular calcification is a common arterial symptom in ESKD patients, who are also prone to uremic conditions. Our previous study showed that uremic serum exposure induced early markers of aSMCs osteogenesis [10]. Therefore, in this study, we examined signaling pathways that may mediate osteogenesis. For example, our previous study indicates that the activation of autophagy can stimulate osteoblast differentiation [18]. In the autophagy pathway, uremic serum exposure increased the expression of *WIPI*, *DFCP1*, *LC3*, *CTSL*, *CTSB*, *CTSD* and *HOPS*. Meanwhile, downregulation of *IGFR*, *PTEN*, *MEK1/2*, *BNIP3*, *MTOR*, *AMPK* and *IP3R* was detected in aSMCs after uremic serum exposure (Supplemental Figure S6). In addition, several important positive transcription factors or growth factors that were able to stimulate osteogenesis were upregulated after uremic serum exposure, including *Runx2*, *DLX5*, *FGF1*, *2* and *7*, *PDGFD*, *VEGFB* and *BMP4*. Furthermore, consistent with the activation of autophagy after uremic serum exposure, our KEGG ORA analysis also showed that the PI3K-AKT signaling pathway was predicted to be negatively enriched, as evidenced by the downregulation of a set of genes, such as *RTK*, *ECM*, *ITGA*, *JAK*, *RAS*, *PP2A*, *PKC*, *MEK*, *PTEN*, *HSP90*, *YWHA* (*14-3-3*) and *CCN* (*cyclin*) (Supplemental Figure S7). However, the downregulation of suppressors of this pathway, such as *PP2A* and *PTEN*, may lead to activation of this pathway, which needs to be confirmed in a future study.

## 4. Discussion

In a prior study of a select group of genes, we identified several important signaling pathways involved in the response of porcine VSMCs to uremic conditions [10]. To further define this complex response, we isolated RNA from UPA and NPA for bulk RNA sequencing. Based on this comprehensive, unbiased approach, we identified 295 genes that were upregulated and 138 genes that were downregulated after exposure to uremic serum. GO MF analysis demonstrated that energy-expenditure-associated pathways and protein post-translation modification pathways were predicted to be negatively enriched, while antioxidant activity, peroxidase activity and glutathione peroxidase activity were predicted to be positively enriched after uremic serum exposure. KEGG pathway mapping of DEGs revealed the specific genes involved in the response to uremic serum exposure, including the cell cycle, cell senescence, cytoskeleton structure regulation, cellular waste disposal and cellular redox homeostasis regulation pathways. These findings suggest that these signaling pathways may be involved in the cellular response to uremic toxin exposure that contributes to the clinical symptoms in CKD/ESKD patients.

Atherosclerosis and calcification are common symptoms in uremic patients, which primarily occur in arteries. Although the alterations of arteries in these patients have been clearly observed, such as fibroelastic intimal thickening and the calcification of elastic lamellae, the causes of these qualitative changes remain to be determined [19,20]. One of our previous studies identified that several key aspects of uremia-induced phenotypic switch were stronger in aSMCs compared to venous VSMCs, including the production of ECM proteins, cellular calcification, and a proinflammatory state [10]. In the current study, using bulk RNA sequencing, we identified additional pathways and genes that might be responsible for these alterations. First, alterations in the expression of genes involved in the cell cycle and cellular senescence suggest these processes are likely to be negatively enriched in aSMCs after exposure to uremic serum. The predicted cell cycle dysregulation is attributed not only to downregulated *CCND*, *CCNB* and *CDK1* but also to upregulated cell cycle inhibitors, such as *CDKN1B* and *1C* (Kip1 and 2), and *ZBTB17* (Miz1). Although this is inconsistent with previous observations that uremic serum stimulates aSMCs proliferation [10,21], our KEGG ORA analysis also indicated that p53 and Hippo signaling pathways, which negatively regulate proliferation, were predicted to be negatively enriched after uremic serum exposure. Therefore, the enhancement of cell proliferation after exposure to uremic serum may be due to the balance of multiple signaling pathways that impact the cell cycle. The suppression of the cellular senescence pathway was also unexpected because CKD and uremic conditions are normally associated with enhanced cellular senescence [22]. However, cellular senescence normally occurs due to persistent stressors, but our current study only examined early cellular responses (120 h after uremic serum exposure) in aSMCs. Therefore, it is possible that cellular senescence is suppressed in the early stages of uremic exposure. Furthermore, the current study only examined transcriptional changes, which may differ from the final outcomes at the protein level and functional level that are controlled by many factors, including translational efficiency, post-translational modifications and cofactor availability. Future studies of specific pathways of interest will be required to determine the complex relationships between the transcriptional changes we identified, pathway activity, and cell behavior.

It is not surprising that cells are under stress when exposed to uremic serum due to the presence of a variety of uremic toxins, such as indoxyl sulfate and p-cresyl sulfate. To adapt to stress, cells may halt energy-intensive processes, such as the cell cycle and protein synthesis, to allow cells to survive [23]. Consistent with this, our analysis demonstrated that the cell cycle, ATP-dependent activity, ATP-hydrolysis activity, protein kinase activity and translation factor activity were predicted to be negatively enriched after uremic serum exposure. Similarly, a previous study used blood cells to show lower mRNA processing and transport, protein transport, chaperone functions, and unfolded protein response in uremia [8]. Our data further suggests that oxidative stress in particular may be important in aSMCs under uremic conditions. Oxidative stress, caused by an imbalance in ROS generation and antioxidant production, oxidizes and damages cellular components, including lipids, proteins and DNA, which are highly associated with the progression of CKD [24,25]. During the progression of CKD, accumulated uremic toxins may therefore exacerbate ROS generation and worsen oxidative damage [26]. Indeed, our current study revealed that aSMCs exposed to uremic serum significantly upregulated the gene expressions that are associated with several cellular redox regulating activities and/or pathways, such as electron transfer activity, glutathione peroxidase activity, antioxidant activity, oxidative phosphorylation, glutathione metabolism and peroxisome signaling pathways. Involved genes include but are not limited to *COX1* and *3*, *ND1-5* (essential genes for complex I assembly in the respiration chain), *CAT, GPX1, GSTK1* (cellular detoxification), *MGST1* and *3* (glutathione-dependent peroxidase activity toward lipid hydroperoxides), *PRDX5, SOD3*, and *PAOX* (producing hydrogen peroxide). These results support the notion that uremic toxins strongly enhance cellular oxidative stress, which may be an effective therapeutic target.

In addition, our study also revealed that ATP-dependent protein folding chaperone activity and protein processing in the ER were predicted to be negatively enriched after uremic serum exposure, which is consistent with previous findings in human blood cells [8]. Inhibiting ATP-dependent machinery often leads to the accumulation of misfolded proteins, which may aggravate diseases [27]. The suppression of protein processing in the ER can initiate an ER stress response, leading to cellular UPR [28]. The UPR triggers protein translation suppression (PERK pathway), mRNA degradation (IRE1 pathway), selective mRNA release, increased misfolded protein degradation via ER-associated degradation (ERAD) and enhanced autophagy to remove accumulated misfolded proteins that cannot be cleared via ERAD [29,30]. Our study demonstrated that the expressions of a group of genes that are associated with these events were altered after uremic serum exposure. The main downregulated genes include chaperone proteins (e.g., *HSPA2*, *HSPA13* and *HSP90AA1*) and protein transporters, such as *Sec62/63* (forming a complex crucial for post-translational protein translocation and ER homeostasis), *Sec23/24* (transporting proteins from the ER to the Golgi apparatus), *ATF6* (a key transmembrane sensor and transcription factor that regulates UPR) and *Ero1* (involved in cell redox homeostasis and chaperone cofactor-dependent protein refolding). The main upregulated gene was *MAN1B1* (*ERmanI*), which is a crucial enzyme in the ERAD pathway [31]. Therefore, ER stress may be another potential therapeutic target for CKD/ESKD patients.

Vascular calcification, which mainly occurs in arteries, is a hallmark of CKD in patients, particularly in later stages, such as in ESKD/uremic patients. Calcific uremic arteriolopathy (calciphylaxis) is a severe form of vascular calcification, which is caused by calcium deposition in the media of the arterioles [32], resulting in skin necrosis and a high mortality rate [33]. Unfortunately, the exact mechanism of calciphylaxis is unclear [34]. Previously, an increase in the calcium–phosphorus product was proposed to cause calcification [35]; however, increasing evidence suggests that calcification involves active cellular processes, not just passive mineralization [36]. One of our prior studies demonstrated that aSMCs are more susceptible to entering calcification programs in response to uremic toxins, as indicated by high protein expression of early osteogenesis markers ALP and Runx2 [10]. Consistently, our current study also showed that Runx2 gene expression in aSMCs was upregulated after uremic serum exposure. In addition, our prior studies demonstrated that the activation of autophagy is required for early stages of osteoblast differentiation [18], and our current study revealed that autophagy was predicted to be positively enriched after uremic serum exposure in aSMCs. Even though the interaction between autophagy and vascular calcification is complicated, and may be affected by the disease stage, anatomical location, and surrounding microenvironment [37], autophagy is considered a novel therapeutic target in vascular calcification [38].

This study revealed altered expression of genes associated with several important signaling pathways after exposure to uremic conditions, which may contribute to artery-specific vascular diseases. However, it is not feasible to simultaneously show all potential pathways that may be involved in causing these diseases. These unmentioned pathways include but are not limited to protein translation, protein kinase activity, IgSF CAM signaling and mitophagy pathways, which were predicted to be negatively enriched, as well as fatty acid degradation and phospholipase D signaling pathways, which were predicted to be positively enriched. In addition, this was a one-time cross-sectional study; thus, all observations only reflect the status of the single time point (120 h after uremic serum exposure) and likely reflect an early stage of uremic condition exposure. A multi-time point assessment of gene expression in uremic conditions should provide important insights into the temporal patterns of the response to uremia. Another limitation of this study arises from the inherent complexity of RNA-seq data analysis and interpretations. Many signaling pathways include both positive and negative feedback loops, and associated genes may positively or negatively regulate the pathway. These “mixed” results complicate our ability to determine the net effect of the changes in gene transcription. More importantly, current bioinformatic analyses seem limited in their ability to disentangle these complex patterns. Furthermore, this study only provides data at the mRNA level, which may differ from the protein level, which is controlled by many factors, such as translation efficiency and post-translational modification. Future functional studies are also required to reveal the precise contribution of these various pathways to the complex response of aSMCs to uremic conditions. Those studies should include assessment of the protein expression and pathway activity changes implicated in this study.

## 5. Conclusions

In summary, our current study identified transcriptional changes in aSMCs under uremic conditions that suggest perturbations in several important cellular processes, including cell cycle regulation, cellular redox homeostasis regulation, cytoskeleton structure regulation, ER stress response and cellular waste disposal. These changes may contribute to artery-specific diseases in CKD/ESKD patients. Using the results of this study as a starting point, future investigations will hopefully identify specific mechanisms of uremia-induced alterations in aSMCs. These mechanistic pathways may result in new biomarkers for risk stratification and potentially druggable targets to ameliorate artery-specific diseases and reduce the risk of CVD and mortality in CKD/ESRD patients.

## Data Availability

The authors declare that raw sequencing data have been submitted to NCBI GEO. All other data supporting the findings of this study will be made available from the corresponding authors upon request.

## Supplementary Materials

The following supporting information can be downloaded at: https://www.mdpi.com/article/10.3390/biom16091252/s1. Figure S1: Workflow of whole project. Figure S2: Cellular senescence pathway map. KEGG cellular senescence signaling pathway rendered with gene-level differential expression mapped onto pathway nodes (Pathview). Node color represents log2 fold change (UPA/NPA) using the displayed scale (green, downregulated in UPA; red, upregulated in UPA). Nodes without color indicate genes not mapped or not present in the input expression contrast. Figure S3: Integrin signaling pathway map. KEGG integrin signaling pathway rendered with gene-level differential expression mapped onto pathway nodes (Pathview). Node color represents log2 fold change (UPA/NPA) using the displayed scale (green, downregulated in UPA; red, upregulated in UPA). Nodes without color indicate genes not mapped or not present in the input expression contrast. Figure S4: Cytoskeleton in muscle cells pathway map. KEGG cytoskeleton in muscle cells pathway with DEG log_2_FC values overlaid (UPA/NPA). Red indicates upregulation in UPA, and green indicates downregulation in UPA. Figure S5: Regulation of actin cytoskeleton pathway map. KEGG regulation of actin cytoskeleton pathway with log2 fold changes (UPA/NPA) projected onto pathway nodes (Pathview). Coloring follows the displayed scale (green, downregulation in UPA; red, upregulation in UPA). Figure S6: Autophagy pathway map. KEGG autophagy pathway with differential expression overlaid as log2 fold change (UPA/NPA) (Pathview). Mapped genes are colored according to the displayed scale (green, downregulation in UPA; red, upregulation in UPA). Figure S7: PI3K-AKT signaling pathway map. KEGG PI3K-AKT pathway with differential expression overlaid as log2 fold change (UPA/NPA) (Pathview). Mapped genes are colored according to the displayed scale (green, downregulation in UPA; red, upregulation in UPA). Table S1: Biochemical parameters of pig serum used in this study (n = 2). Table S2: Uremic toxin contents in the pig serum used in this study (n = 2).

## Abbreviations

The following abbreviations are used in this manuscript:

ESKD	End-stage kidney disease
aSMCs	Arterial smooth muscle cells
CKD	Chronic kidney disease
DEGs	Differentially expressed genes
Log2FC	Log2 fold change
FDR	False discovery rate
Padj	Adjusted *p*-value
pCut	*p*-value cutoff
ER	Endoplasmic reticulum
UPR	Unfolded protein response
ERAD	ER-associated degradation
ROS	Reactive oxygen species
GSH	Reduced glutathione
CVD	Cardiovascular diseases
VSMCs	Vascular smooth muscle cells
NPA	Normal pig serum treated aSMCs
UPA	Uremic pig serum treated aSMCs
Go	Gene Ontology
ORA	Overrepresentation analysis
GSEA	Gene set enrichment analysis
MF	Molecular function
ECM	Extracellular matrix
