# Supplementary material for: Uremic Serum Alters Gene Expression Profiles and Signaling Pathway Activity in Porcine Arterial Smooth Muscle Cells
Source: Biomolecules. 2026 Aug 28;16(9):1252. doi: 10.3390/biom16091252 (PMC13604813; doi:10.3390/biom16091252)
Supplement: Supplementary file 1 [file biomolecules-16-01252-s001.zip › biomolecules-4457096-supplementary.pdf]

Supplementary Materials

Table S1. Biochemical parameters of pig serum used in this study (n = 2).

Biochemical parameters	Normal Pig Serum				Uremic Pig Serum				Fold Increase
Animal ID	72C	82C	Avg	SD	64C	68C	Avg	SD	
Blood Urea Nitrogen (BUN) (mg/dl)	8.0	9.0	8.5	0.7	43.0	50.0	46.5	4.9	5.5
Creatinine (CRE) (mg/dl)	1.7	1.4	1.6	0.2	9.5	8.6	9.1	0.6	5.8
Albumin (ALB) (g/dl)	4.1	4.4	4.3	0.2	3.6	3.3	3.5	0.2	0.8
Alkaline Phosphatase (ALP) (U/L)	92	135	113.5	30.4	149	183	166.0	24.0	1.5
Alanine Aminotransferase (ALT)(U/L)	28	33	30.5	3.5	26	31	28.5	3.5	0.9
Calcium (mg/dl)	10.8	10.9	10.9	0.1	10.5	11.1	10.8	0.4	1.0
Phosphate (mg/dl)	10.1	9.2	9.7	0.6	9	8.5	8.8	0.4	0.9
Glucose (mg/dl)	63	107	85.0	31.1	88	82	85.0	4.2	1.0
Na+ (mmol/L)	140	139	139.5	0.7	140	144	142.0	2.8	1.0
K+ (mmol/L)	5.7	5.1	5.4	0.4	5.9	6.4	6.2	0.4	1.1
Total Protein (TP) (g/dl)	5.9	6.6	6.3	0.5	5.6	6.3	6.0	0.5	1.0

Table S2. Uremic toxin contents in the pig serum used in this study (n = 2).

Biochemical parameters	Normal Pig Serum				Uremic Pig Serum				Fold Increase
Animal ID	72C	82C	Avg	SD	64C	68C	Avg	SD	
Hippurate	0.4062	0.4493	0.4062	0.0304	6.5180	5.8856	6.2018	0.4472	15.3
Indoxyl sulfate	0.6513	0.5983	0.6513	0.0375	4.9379	4.2633	4.6006	0.4770	7.1
Kynurenate	0.0001	0.0001	0.0001	0.0000	0.0014	0.0024	0.0019	0.0007	16.7
p-cresol sulfate	0.1363	0.1292	0.1363	0.0051	1.1458	2.0266	1.5862	0.6228	11.6
Phenol sulfate	0.0033	0.0027	0.0033	0.0004	0.1061	0.1856	0.1459	0.0562	44.6
p-cresol glucuronide	1.4373	1.4009	1.4373	0.0257	4.7352	5.1005	4.9178	0.2583	3.4
Phenylacetylglycine	1.0093	0.8875	1.0093	0.0861	4.5026	4.1335	4.3181	0.2610	4.3
Phenol glucuronide	0.0047	0.0044	0.0047	0.0002	0.0391	0.0468	0.0430	0.0054	9.1
4-Ethylphenylsulfate	0.1322	0.1236	0.1322	0.0061	1.3522	1.6948	1.5235	0.2423	11.5
Kynurenine	0.0908	0.0818	0.0908	0.0064	0.1574	0.1533	0.1554	0.0029	1.7

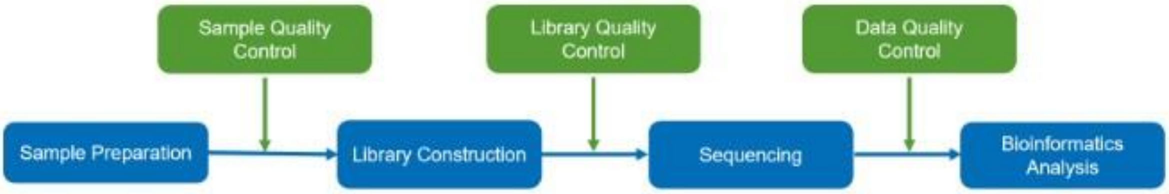

Figure S1. Workflow of whole project.

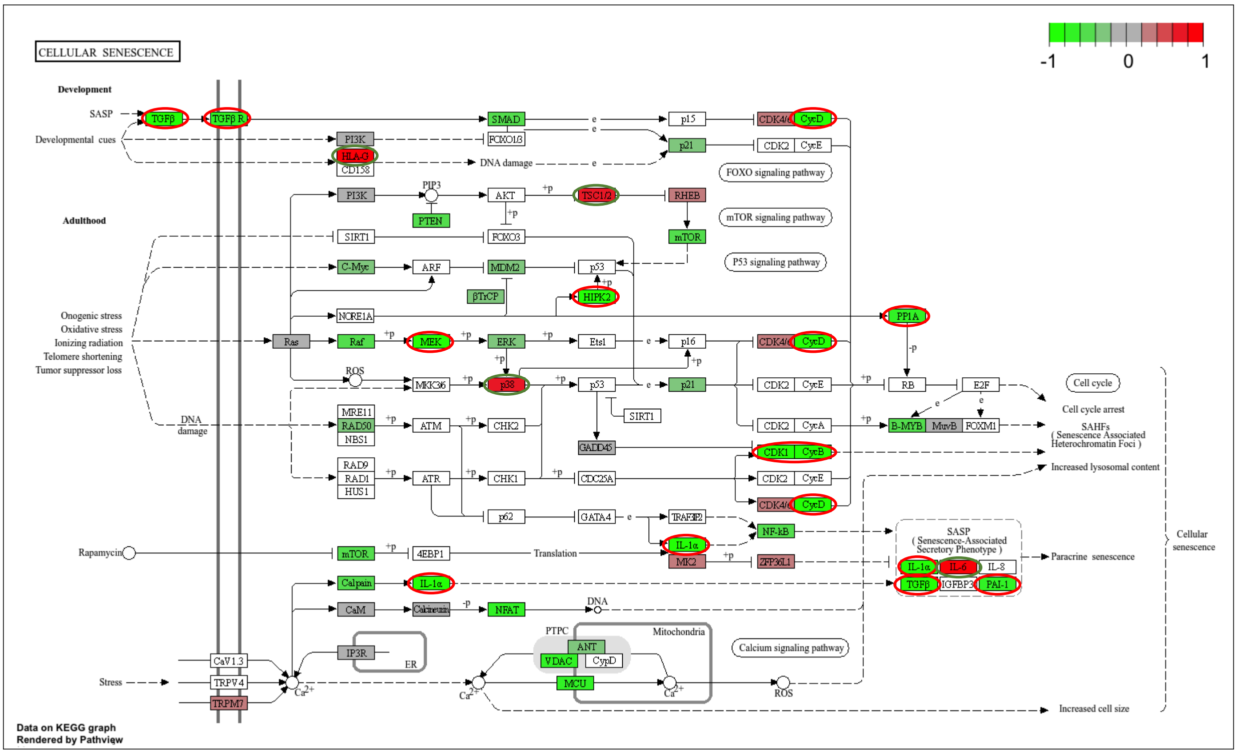

Figure S2. Cellular senescence pathway map. KEGG cellular senescence signaling pathway rendered with gene-level differential expression mapped onto pathway nodes (Pathview). Node color represents log2 fold change (UPA/NPA) using the displayed scale (green, downregulated in UPA; red, upregulated in UPA). Nodes without color indicate genes not mapped or not present in the input expression contrast.

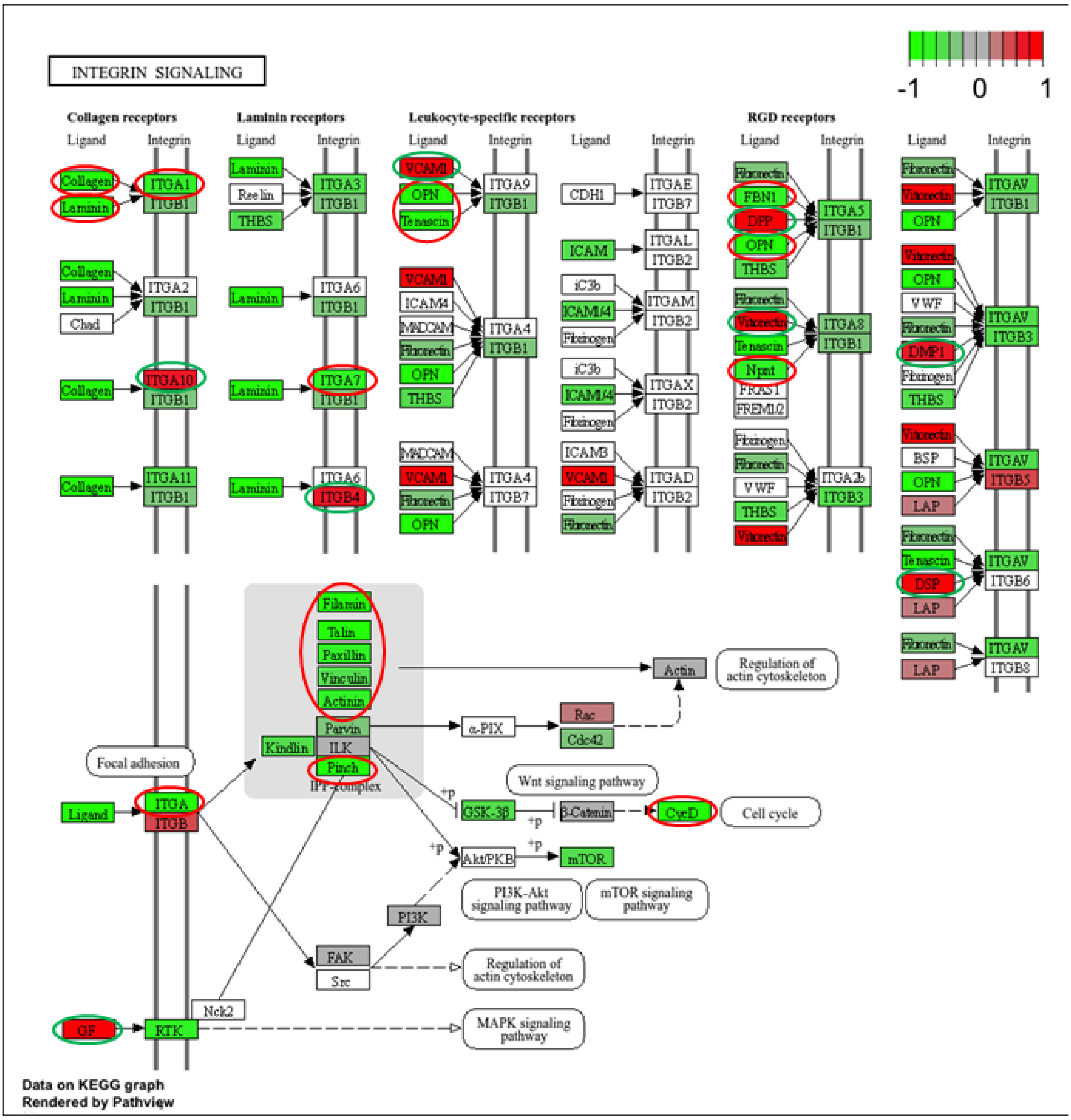

Figure S3. Integrin signaling pathway map. KEGG integrin signaling pathway rendered with gene-level differential expression mapped onto pathway nodes (Pathview). Node color represents log2 fold change (UPA/NPA) using the displayed scale (green, downregulated in UPA; red, upregulated in UPA). Nodes without color indicate genes not mapped or not present in the input expression contrast.

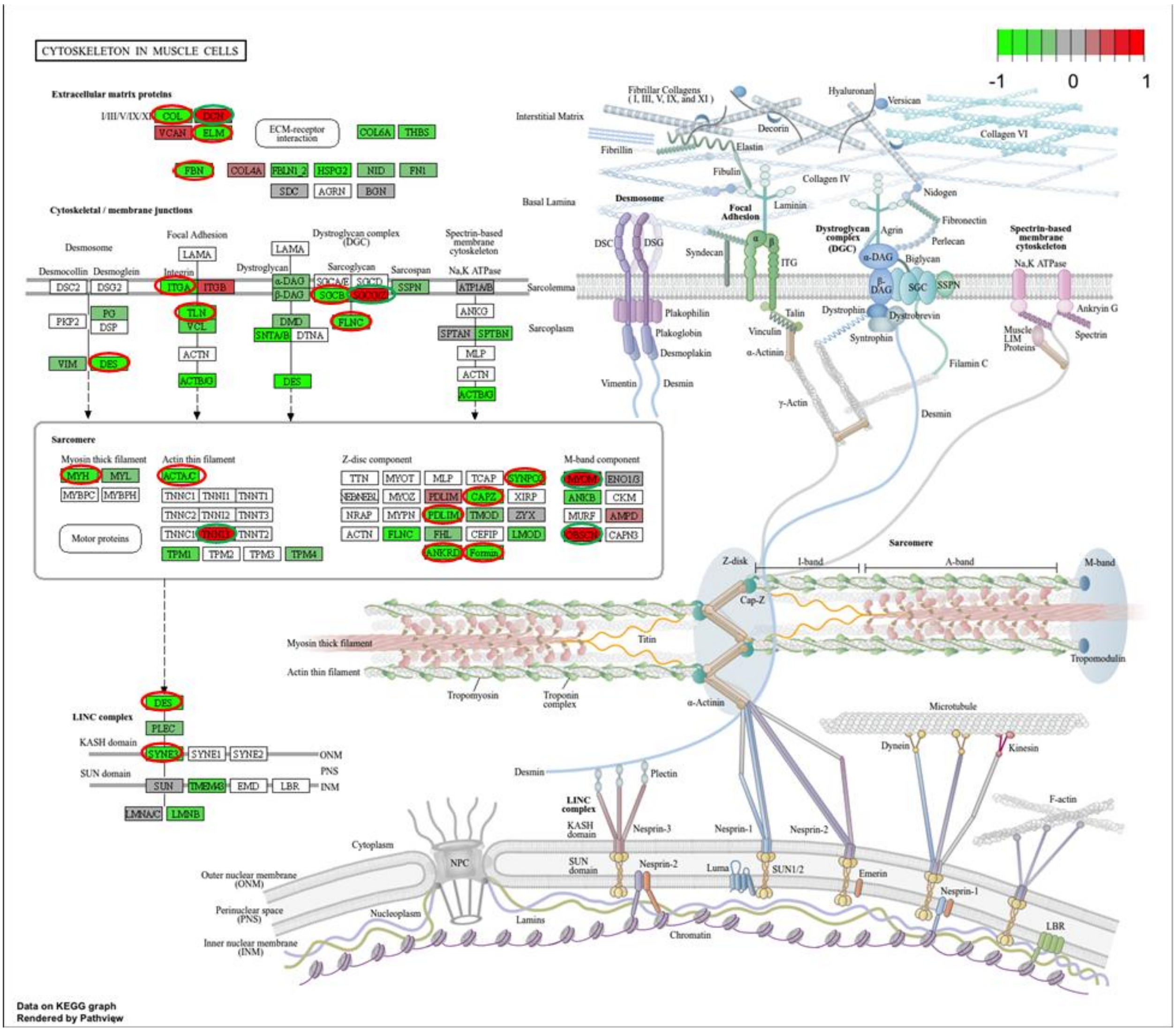

Figure S4. Cytoskeleton in muscle cells pathway map. KEGG cytoskeleton in muscle cells pathway with DEG log_2FC values overlaid (UPA/NPA). Red indicates upregulation in UPA, and green indicates downregulation in UPA.

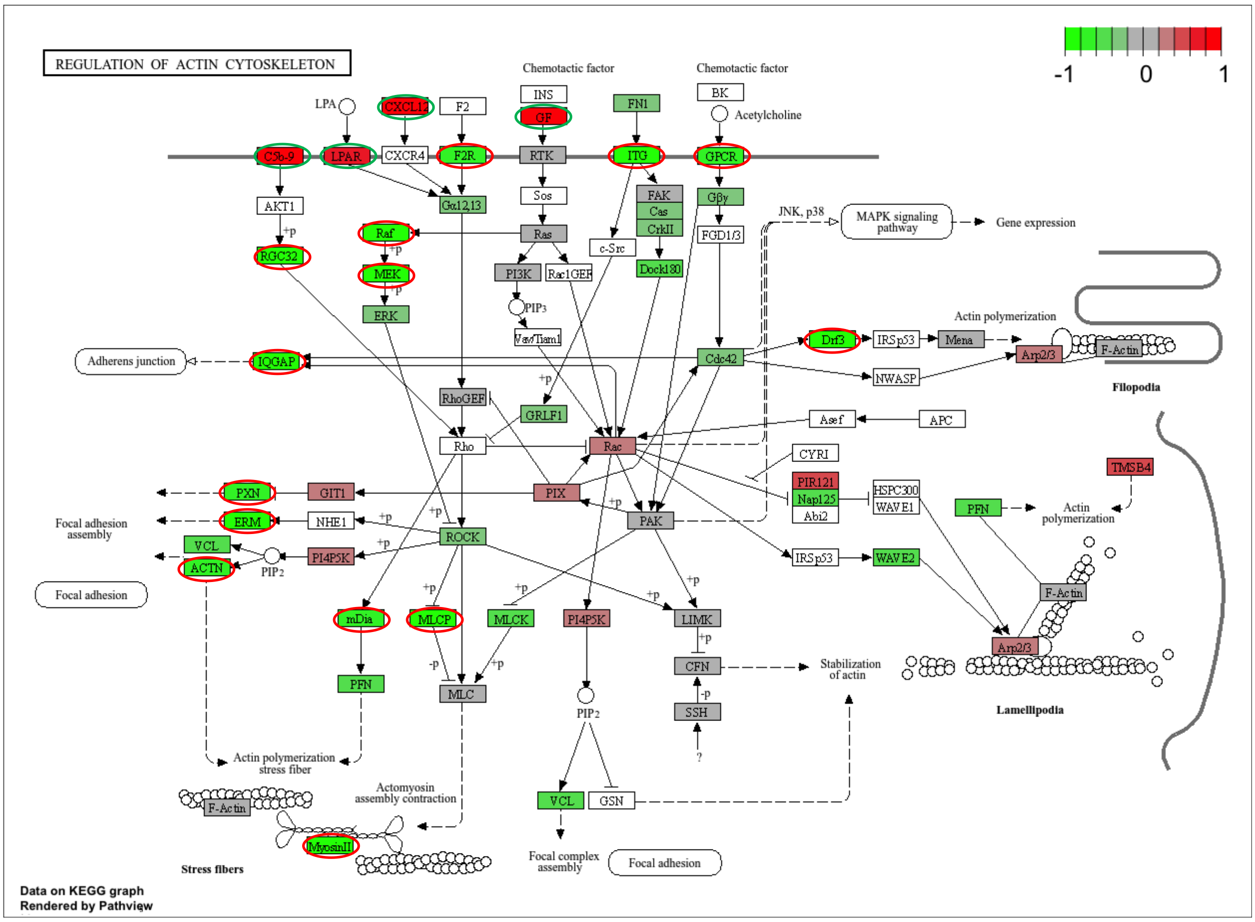

Figure S5. Regulation of actin cytoskeleton pathway map. KEGG regulation of actin cytoskeleton pathway with log2 fold changes (UPA/NPA) projected onto pathway nodes (Pathview). Coloring follows the displayed scale (green, downregulation in UPA; red, upregulation in UPA).

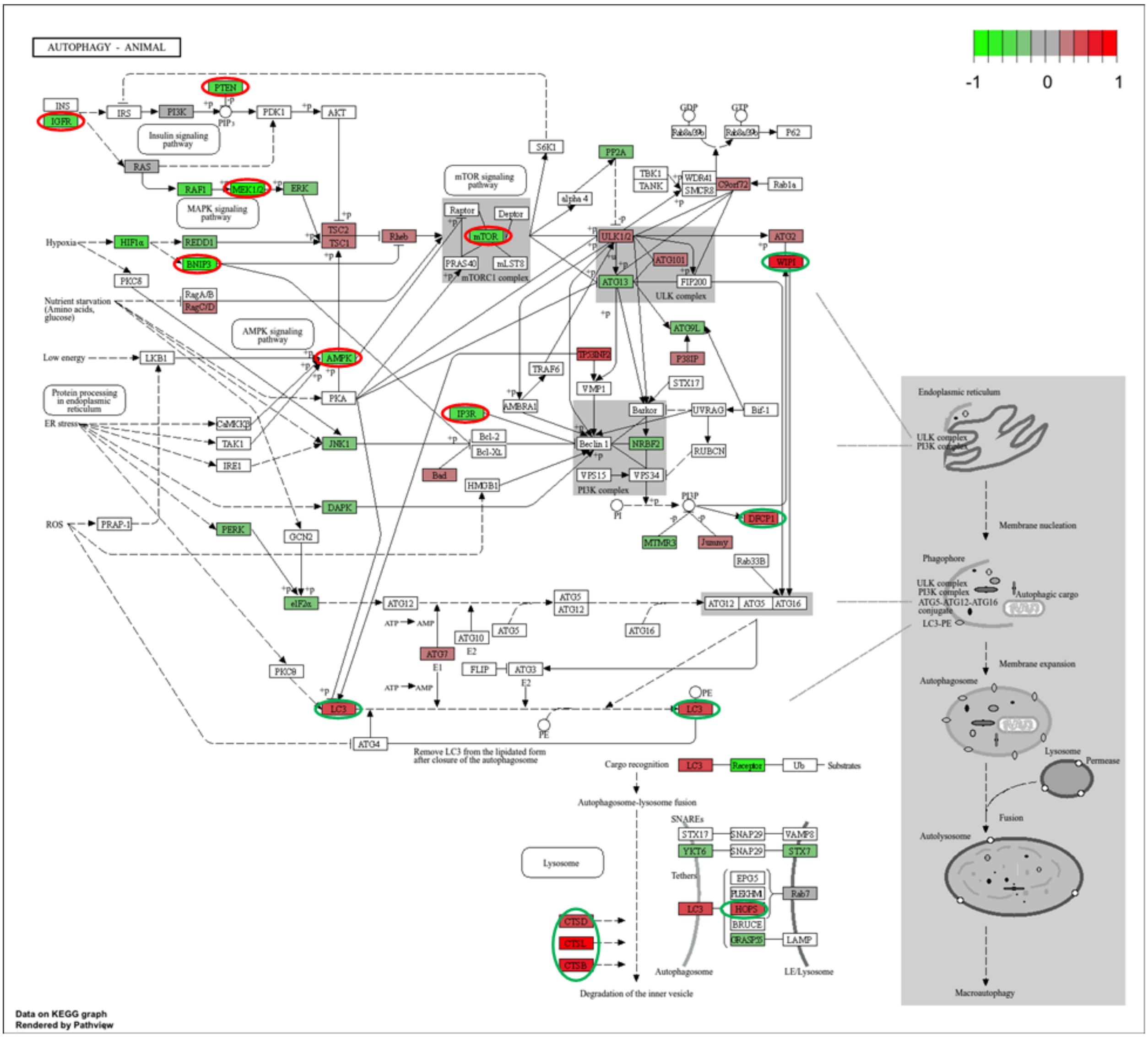

Figure S6. Autophagy pathway map. KEGG autophagy pathway with differential expression overlaid as log2 fold change (UPA/NPA) (Pathview). Mapped genes are colored according to the displayed scale (green, downregulation in UPA; red, upregulation in UPA).

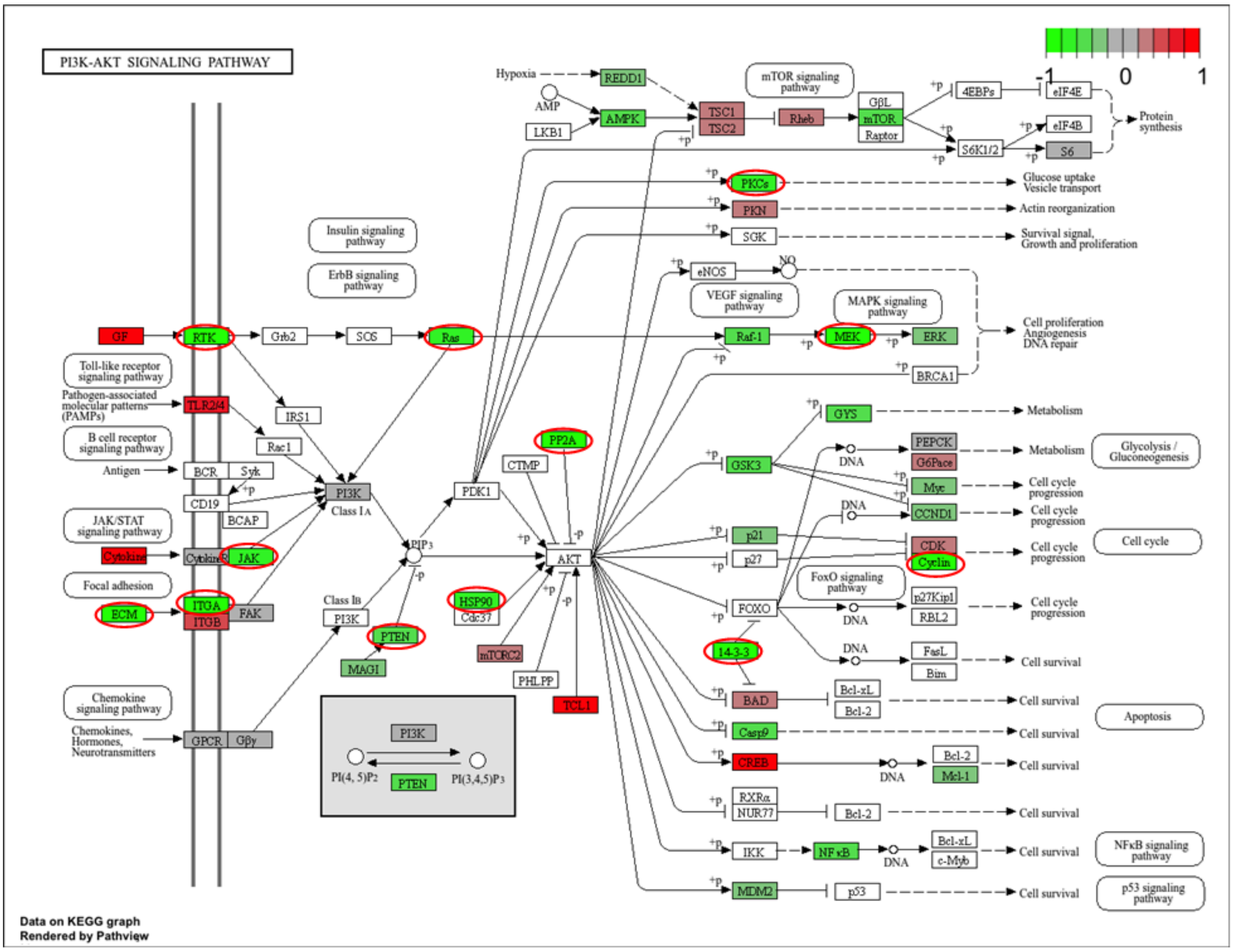

Figure S7. PI3K-AKT signaling pathway map. KEGG PI3K-AKT pathway with differential expression overlaid as log2 fold change (UPA/NPA) (Pathview). Mapped genes are colored according to the displayed scale (green, downregulation in UPA; red, upregulation in UPA).
